# Assessing the Effects of Light on Differentiation and Virulence of the Plant Pathogen *Botrytis cinerea:* Characterization of the *White Collar* Complex

**DOI:** 10.1371/journal.pone.0084223

**Published:** 2013-12-31

**Authors:** Paulo Canessa, Julia Schumacher, Montserrat A. Hevia, Paul Tudzynski, Luis F. Larrondo

**Affiliations:** 1 Departamento de Genética Molecular y Microbiología, Facultad de Ciencias Biológicas, Pontificia Universidad Católica de Chile, Santiago, Chile; 2 Institut für Biologie und Biotechnologie der Pflanzen, Westf. Wilhelms-Universität Münster, Münster, Germany; Oregon State University, United States of America

## Abstract

Organisms are exposed to a tough environment, where acute daily challenges, like light, can strongly affect several aspects of an individual's physiology, including pathogenesis. While several fungal models have been widely employed to understand the physiological and molecular events associated with light perception, various other agricultural-relevant fungi still remain, in terms of their responsiveness to light, in the dark. The fungus *Botrytis cinerea* is an aggressive pathogen able to cause disease on a wide range of plant species. Natural *B. cinerea* isolates exhibit a high degree of diversity in their predominant mode of reproduction. Thus, the majority of naturally occurring strains are known to reproduce asexually via conidia and sclerotia, and sexually via apothecia. Studies from the 1970′s reported on specific developmental responses to treatments with near-UV, blue, red and far-red light. To unravel the signaling machinery triggering development – and possibly also connected with virulence – we initiated the functional characterization of the transcription factor/photoreceptor BcWCL1 and its partner BcWCL2, that form the White Collar Complex (WCC) in *B. cinerea*. Using mutants either abolished in or exhibiting enhanced WCC signaling (overexpression of both *bcwcl1* and *bcwcl2*), we demonstrate that the WCC is an integral part of the mentioned machinery by mediating transcriptional responses to white light and the inhibition of conidiation in response to this stimulus. Furthermore, the WCC is required for coping with excessive light, oxidative stress and also to achieve full virulence. Although several transcriptional responses are abolished in the absence of *bcwcl1*, the expression of some genes is still light induced and a distinct conidiation pattern in response to daily light oscillations is enhanced, revealing a complex underlying photobiology. Though overlaps with well-studied fungal systems exist, the light-associated machinery of *B. cinerea* appears more complex than those of *Neurospora crassa* and *Aspergillus nidulans.*

## Introduction

Light is a strong environmental cue capable of modulating major aspects of the physiology of an organism. Development, metabolism and other complex genetic programs are affected by light in filamentous fungi. Moreover, the effect of light also implies that its absence – darkness – can trigger a variety of fundamental processes, including the mode of reproduction [1], [2]. At the molecular level, these responses have been studied in detail in only a few fungal models such as *Neurospora crassa*, *Aspergillus nidulans*, *Trichoderma reesei* and *Phycomyces blakesleeanus* (reviewed by [3]–[6]). Despite the similarities between the basic mechanisms involved in light perception in these models, important differences have been observed when characterizing photoresponses in these and other fungi, granting the need to investigate – instead of just extrapolating – light-signaling mechanisms in unexplored fungal systems.

The molecular characterization of fungal (blue) light perception was initiated in *N. crassa*, with the genetic isolation of the blue-light receptor *wc-1* (*white collar-1*) [7]. In this organism, light regulates the circadian clock, triggers mycelial carotenoid biosynthesis, conidiation and promotes the formation of protoperithecia [8]. WC-1 is a GATA-type zinc finger transcription factor (TF), containing a DNA binding domain, two PAS (PER-ARNT-SIM) domains involved in protein-protein interactions, two putative transcriptional activation domains, a nuclear localization signal (NLS) and a LOV domain (a specialized type of PAS domain) involved in environmental sensing of light, oxygen and voltage. The chromophore FAD, accommodated in the LOV domain, is essential for the photoreceptor activity of WC-1, and for the light-activation of the WCC [7], [9]. In *N. crassa*, approximately 3% (314 genes) of the transcriptional units exhibit early (observed after 15–30 min) or late (after 60 min and over) light responses as part of a transcriptional cascade initiated by WC-1 [10]. Together with WC-2, another GATA-type TF, WC-1 forms the so-called White Collar Complex (WCC) that directly activates the expression of early light-responsive genes upon light stimulation, among which 24 encode for putative TFs [11]. This group of light-/WCC-dependent TFs include those regulating asexual development (conidiation) such as FL (*fluffy*), SAH-1 (*short aerial hyphae-1*) and CSP-1 (*conidiation separation-1*), sexual development (formation of perithecia) such as SUB-1 (*submerged protoperithecia*) and BEK-1 (*beak-1*) or both processes such as ADV-1 (*arrested development-1*) [12]. In addition, the WCC is a key component of the circadian system, regulating the daily expression of the *frequency* (*frq*) gene, a central component of the transcriptional translational feedback loop that gives rise to the circadian oscillator or pacemaker [13]–[15].

Several other photoreceptors have been identified in fungal systems. They include cryptochromes (UV/blue light receptors), opsins (putative green light receptors), red light sensors known as phytochromes and VIVID orthologs (blue light receptors) [3], [4] which have been shown in one or other fungal system to modulate a variety of processes in response to different light wavelengths [2], [3], [5], [6], [16]. Interestingly, while several filamentous fungi contain orthologs of WC-1 and WC-2, the presence and number of other photoreceptors varies among them, possible reflecting adaptations to different ecological niches. Thus, while in *N. crassa* the small LOV-domain-containing protein VIVID [17] serves a key role in photoadapting the WCC-dependent responses [18]–[20], it is absent in other WCC-containing organisms like *A. nidulans*. Likewise, although red-light responses have been clearly characterized in *A. nidulans* and recently also in *Aspergillus fumigatus* [21]–[24], the deletion of the two phytochromes or the presence of responses to red-light, have not been associated with any phenotypical or molecular changes in *N. crassa* [25].

Only recently light has been recognized as an important modulator of fungal pathogenesis [26], and syndicated as a relevant variable with the potential to affect the outcome of the plant-pathogen interaction by modulating either plant defense responses, virulence of the pathogen or both (reviewed in [27]). Supporting this concept, WC-1 orthologs have been implicated in modulating virulence, but their precise function differs among fungi-host interactions. Thus, involvement of WC-1 orthologs in virulence have been shown for the human pathogen *Cryptococcus neoformans* [28] and in *Magnaporthe oryzae,* the causal agent of the rice blast disease. In the latter fungus, constant light suppresses disease development which is mediated via MGWC-1 [29]. In *Cercospora zeae-maydis,* a plant pathogen that infects leaves through stomata, WC-1 is required for stomata tropism, and for appressorium and lesion formation in maize [30]. Interestingly, in the opportunistic human pathogen *Fusarium oxysporum*, WC-1 is required for causing disease in immunocompromised mice but is dispensable for causing vascular wilt in plants [31].

*Botrytis cinerea* is an important necrotrophic plant pathogen causing the grey mould disease in a variety of dicotyledonous plant species including economically relevant crop plants [32]. Major sources of infection are the air-borne macroconidia that are formed on branched conidiophores at the end of the infection cycle when the fungus has colonized the host and macerated the plant tissue [33], [34]. Under given conditions, *B. cinerea* forms sclerotia that may act as survival structures germinating to produce mycelia and conidia (asexual reproduction) or as “female parent” during sexual reproduction. In the latter case, sclerotia are fertilized by microconidia of the opposite mating type bearing later the apothecia containing the sexual spores [35], [36]. Several reports have been published describing responses of *B. cinerea* strains to different light conditions. These include phototropic responses of conidiophores, conidial germ tubes and apothecia. Moreover, the mode of (asexual) reproduction is determined by light and its absence, respectively (photomorphogenesis). Thus, *B. cinerea* forms conidia in the light and sclerotia in the darkness, and also the differentiation of apothecia on the fertilized sclerotia requires light. Studies undertaken in the 1970′s reported on morphological changes in response to different wavelengths of light, i.e. to near-UV, blue, red and far-red light suggesting the involvement of several photoreceptors in regulating the differentiation of reproductive structures in *B. cinerea* [37], [38].

To unravel the molecular basis of photoreception in *B. cinerea* and its possible impact on virulence, we initiated an approach to study light signaling in this organism. First, to address the high genetic variation that exists among *B. cinerea* isolates, we verified that the commonly used strain B05.10 is an adequate model for studying light responses. Using this genetic background, we investigated the function of the *white collar*-like complex (BcWCL1/BcWCL2) by using mutants deleted for the blue light-sensing BcWCL1 and mutants simultaneously overexpressing both TFs. By this, we demonstrated that the WCC mediates gene expression in response to white light, functions as a repressor of conidiation in the presence and absence of light, and it is required for tolerating excessive illumination and to achieve full virulence in the presence of light. Importantly, we observed that the absence of *bcwcl1* leads to enhanced phenotypic responses to light, which, in addition to gene expression data, indicates that a complex biology underlies photoresponses in *B. cinerea*.

## Materials and Methods

### *B. cinerea* strains

Strain B05.10 of *B. cinerea* Pers. Fr. [*Botryotinia fuckeliana* (de Bary) Whetzel] was isolated from *Vitis vinifera* (Germany) and is used as the recipient strain for genetic modifications [39], [40]. Strains T4 and 1787 were isolated from tomato (France) and strawberry (Japan), respectively [41], [42]. Other *B. cinerea* strains screened in this study were isolated from *V. vinifera* and strawberry in Germany in the 1990′s [40]. Genome sequences of strains B05.10 and T4 were published [43] and recently updated [44], and are available at URGI and BROAD Institute websites (http://urgi.versailles.inra.fr and http://www.broadinstitute.org/, respectively).

### Culture conditions

*B. cinerea* strains were cultivated in Petri dishes containing one of the following solidified media: synthetic complete medium (CM) [45], potato dextrose agar (PDA, AppliChem) with and without 10% homogenized bean leaves, Gamborg B5 (Duchefa Biochemie) supplemented with 2% glucose, or synthetic minimal medium (MM) (modified Czapek Dox containing 2% sucrose, 0.1% KH_2_PO_4_, 0.3% NaNO_3_, 0.05% KCl, 0.05% MgSO_4_×7 H_2_O, pH 5). The strains were also cultivated in PDA-containing hollow-glass tubes, known as race tubes, covered with sterile hydrophobic cotton at both ends. The strains were incubated at 20°C using Percival incubators equipped with cool white light fluorescent tubes (light intensity up to 100 micromoles/m^2^/s; wavelength 400–720 nm) in a 12:12 h light:dark regime (LD). When indicated, strains were subjected to constant light (LL) or dark (DD) conditions, or light of different wavelengths using Roscolux color filters (Rosco Laboratories Inc.). Theses filters include Roscolux #15 (deep straw), #27 (med red), #312 (canary), #381 (baldassari blue) and #389 (chroma green). Light filter transmission spectrums are indicated when needed (see below). For light pulse experiments, cultures were first grown in the dark (DD) for 48 h and then exposed to light for the indicated periods of time.

### RNA extraction and Real-time quantitative RT-PCR (RT-qPCR)

For RNA isolation, mycelia were obtained from cellophane-covered solid media (PDA or CM). DD cultures were harvested in a temperature-controlled darkroom equipped with low-intensity red-safety lights, and immediately frozen in liquid nitrogen. Samples from LL or light pulse culture conditions were harvested under white light, and processed accordingly. All samples were kept at −80°C until further purification. Frozen mycelia were ground to powder, and total RNA was isolated using TRIzol reagent (Invitrogen) as described by [10]. Total RNA quantity and quality was verified using NanoDrop (Thermo Scientific) and by electrophoresis in a formaldehyde-containing agarose gel (1.2% w/v). RNA was further purified using the RQ1 RNase-free DNase (Promega), following the manufacturer's instructions. Absence of genomic DNA contaminations in the samples was confirmed by RT-minus reactions (data not shown). Thereafter, RNA samples (1 µg) were reverse transcribed using the MMLV reverse transcriptase (Promega), according to manufacturer's directions. One µl of cDNA was used in each RT-qPCR reaction.

RT-qPCRs procedures were conducted according to the MIQE guideline (Minimum Information for Publication of Quantitative Real-Time PCR Experiments) [46]. Transcript quantification was achieved using the SensiMixPlus SYBR Green kit (12.5 µl reactions; Quantace) and the LightCycler 480 detection system (Roche) using the LightCycler 480 software (version 1.5.0.39), as described in manufacturers' manuals. Primer sequences and predicted Tm values, as well as amplicon lengths, are shown in Table S1. The RT-qPCR was performed as follows: 10 min at 95°C followed by 40 cycles of 15 s at 95°C, 15 s at 58°C or 60°C (see Table S1) and 15 s at 72°C, followed by a melting cycle from 55 to 95°C to check for amplification specificity. C_q_ values were acquired during the annealing period of the RT-qPCR. Standard quantification curves with several serial 10-fold dilutions of RT-qPCR products were employed to calculate the amplification efficiency (E) of each gene, according to the equation E =  [10^(1/slope)^]-1. The obtained E values are also shown in Table S1. These values were used to obtain a more accurate ratio between the gene of interest (GOI) and the expression of the reference genes (*actin* and *elongation factor 1 beta*; BC1G_08198 and BC1G_03337, respectively) employed for normalization. Accurate normalization of RT-qPCR data was achieved by geometric averaging of two internal reference genes (NF: normalization factor; [47]). In all experiments, expression values are referred to the culture grown in DD.

### Northern blot hybridizations

Samples (25 μg) of total RNA, prepared as described above, were transferred to Hybond-N+ membranes after electrophoresis on a 1% (w/v) agarose gel containing formaldehyde, according to standard methods [48]. Blot hybridizations with random-primed α-^32^P-dCTP-labelled probes were performed as described previously [49].

### Cloning of *bcwcl1* replacement cassettes

Two *bcwcl1* replacement cassettes were assembled using yeast recombinational cloning (YRC) as described previously [50]. These cassettes are referred herein as replacement cassette A and B (Figure S1A). Thus, the 5′- and 3′-non-coding regions of *bcwcl1* were amplified from genomic DNA of *B. cinerea* B05.10 using the primer pairs indicated in Table S2. The hygromycin (*hph*) resistance cassette was amplified from vector pLOB1 [51] (Δ*bcwcl1*, mutant 1, replacement cassette A) or pCSN44 (obtained from the Fungal Genetics Stock Center, [52]) as template (Δ*bcwcl1*, mutant 2 and 3; replacement cassette B). Primers employed for these PCR reactions contained 30-bp-overlapping regions, thus allowing homologous recombination. Detailed descriptions of the vectors containing the *bcwcl1* replacement cassettes are indicated in Figure S1. Fragments were co-transformed with the linearized pRS426 vector [53] into uracil-auxotrophic *Saccharomyces cerevisiae* strain FY834 [54] for assembly. After yeast transformation, the plasmid containing the construct was recovered from yeast by *Escherichia coli* (DH5α) transformation. Junctions were sequenced to confirm the absence of mutations (data not shown). Thereafter, the replacement cassettes were amplified using universal primers flanking the recombination region chosen in pRS426 (Table S2) and the Expand High Fidelity DNA polymerase (Roche) and used for transformation of *B. cinerea*.

### Cloning of *bcwcl1* complementation cassette

For this purpose, the open reading frame (ORF) of *bcwcl1* was amplified from genomic DNA using the primer pair *bcwcl1*-P*oliC*-F/T*gluc*-R (Table S2) and assembled with the NcoI/NotI-digested plasmid pNDN-OGG [55] by YRC, yielding pNDN-*bcwcl1.* This vector contains *bcwcl1* under control of the *oliC* promoter from *A. nidulans* (P*oliC*) and the glucanase terminator of *B. cinerea* (T*gluc*), a nourseothricin resistance cassette and flanking sequences for facilitating the targeted integration at the *bcniaD* locus (nitrate reductase).

### Generation of *bcwcl1* deletion and complemented mutants

Protoplast generation and transformation were performed as described previously [55]. Succinctly, *B. cinerea* spores from a one-week old culture (Petri dish) were incubated for 18 h at 20°C and 120 rpm in 100 ml malt extract medium (1.5%). Protoplasts from the young mycelia were generated using an enzymatic mixture containing Lysing enzyme (Sigma) and Yatalase (Takara). Protoplasts of wild-type B05.10 strain were mixed with 30 µl of purified PCR products (replacement cassette A and B, respectively) (in PEG solution; 25% PEG 3350, CaCl_2_ 1M, Tris-HCl 1M, pH 7.5) and following a regeneration step of 20–24 h, they were overlaid with SH agar containing 70 µg/ml hygromycin B (Invitrogen). Homokaryotic derivates were achieved by spreading conidial suspensions on Gamborg B5–2% glucose supplemented with 70 µg/ml hygromycin B and subsequent transfer of single colonies to new Petri dishes. Following DNA extraction [56], transformants that have undergone homologous integration at *bcwcl1* were confirmed by PCR using locus-specific primers (oL588, oL589) in combination with ones binding in the hygromycin resistance cassettes (oL584, oL585 and oL29, oL32 for replacement fragments A and B, respectively). Absence of wild-type alleles was confirmed by using primers oL586 and oL587 designed to the substituted region of *bcwcl1* (Figure S1). PCR-verified mutants were further analyzed by means of Southern blot hybridization employing the DIG Easy Hyb Hybridization solution and the PCR DIG Probe Synthesis Kit (Roche) following the manufacturer's directions. Diagnostic PCRs and Southern blot analysis are shown in Figure S1.

For complementation experiments, protoplasts of the deletion mutant (Δ*bcwcl1*-1) were transformed with the linearized vector pNDN-*bcwcl1* and overlaid with SH agar containing 140 µg/ml nourseothricin (Werner-Bioagents, Germany). Targeted integration of the construct at *bcniaD* was detected using the locus-specific primer oL1716 and primer oL1226, which binds to the nourseothricin resistance cassette and to *bcwcl1*, respectively.

### Virulence assays

Assays were conducted essentially as described previously [55]. Briefly, conidia were suspended in Gamborg B5 medium supplemented with 2% glucose and adjusted to a final concentration of 2×10^5^ conidia/ml in Gamborg B5 and 10 mM KH_2_PO_4_/K_2_HPO_4_, pH 6.4. Conidial suspensions (7.0 µl) were used to inoculate leaves of *Arabidopsis thaliana* (accession Col-0) of approximately four-week-old or primary leaves of French bean (*Phaseolus vulgaris* cv. 90598). All plants were incubated inside plastic boxes at 20°C under humid environment, in LL, LD or DD conditions, within Percival I-30BLL incubators, for the indicated periods of time. Lesions on bean leaves were measured manually, while those on *A. thaliana* leaves were recorded semi-automatically using the ImageJ software using an external calibration scale.

### Trypan blue staining

For detection of *B. cinerea* hyphae in infected plant tissues, detached *A. thaliana* leaves were washed for 1 h with gentle agitation in absolute ethanol at 60°C to remove chlorophyll. Thereafter, leaves were incubated for 30 min in lactophenol trypan blue solution (water: glycerol: lactic acid (1:1:1) +10 µl of trypan blue solution 25 mg/ml (Sigma-Aldrich, Germany)). Finally, stained leaves were incubated for 20 min with gentle agitation in a destained solution (water: glycerol: lactic acid (1:1:1)) and transferred into 50% glycerol solution for microscopy.

### DAB staining

H_2_O_2_ levels *in planta* were determined employing 3,3′-diaminobenzidine (DAB) staining as described [57]. Briefly, detached leaves were incubated in a 2 mM EDTA solution pH 5.5, and subsequently incubated in a 5 mM DAB solution pH 3.8, for 2 h with gentle agitation. Leaves were destained in lactophenol. Thereafter, images were acquired using a Nikon Eclipse 80*i* microscope attached to a digital camera.

## Results

### Some but not all *B. cinerea* wild isolates exhibit phenotypic responses to light

**Figure 1:**
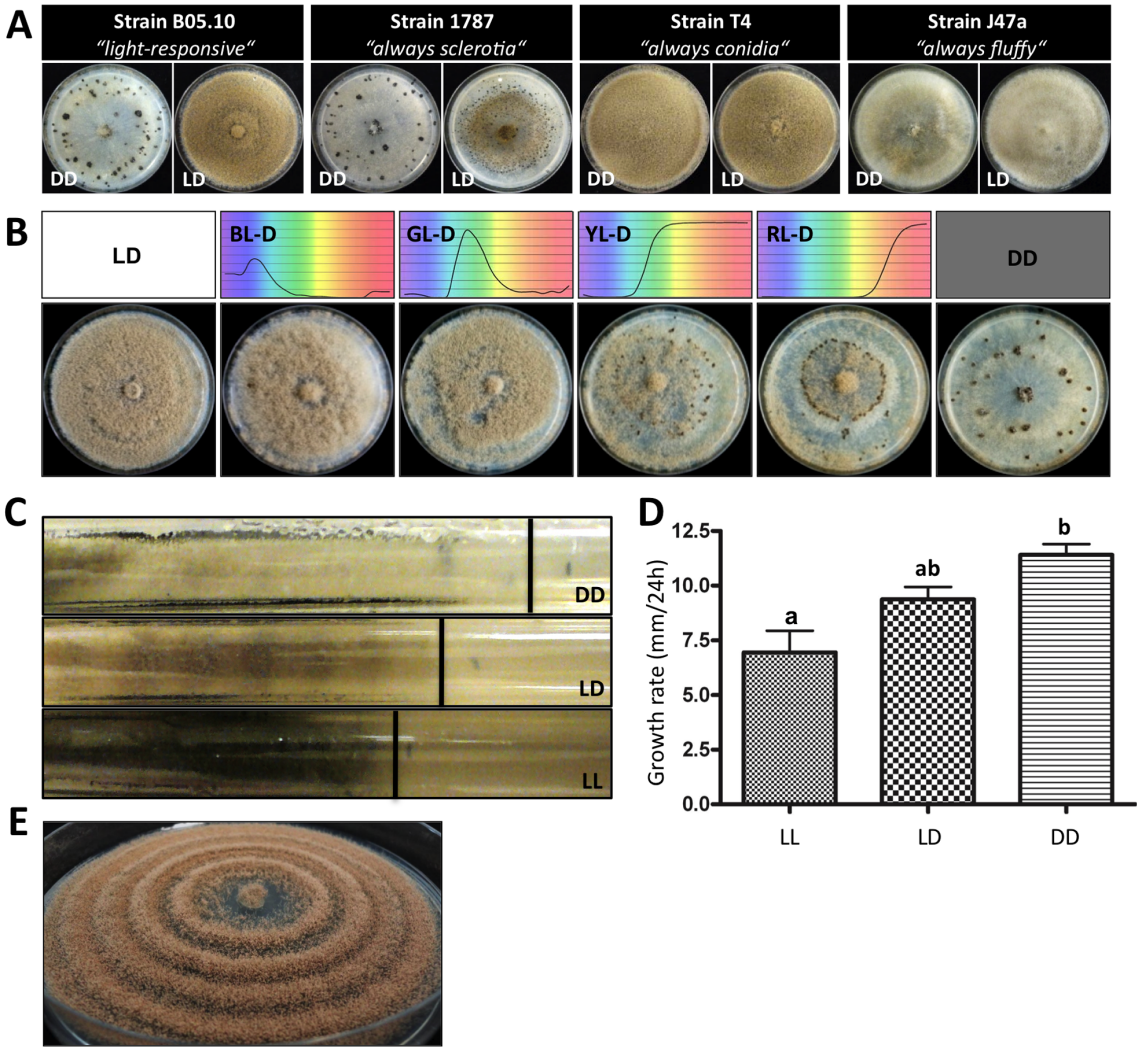
Light controls differentiation and growth in most but not all analyzed *B. cinerea* strains. (**A**) Photomorphogenic developmental programs observed for *B. cinerea* strains. Strains B05.10, 1787, T4 and J47a were incubated on solid complete medium (CM) for 14 d in LD (12:12 h light:dark) or DD (constant darkness) conditions. Strain B05.10 responds to light, and produces conidiophores and conidia in LD and sclerotia in DD. Always sclerotia, conidia and “*fluffy”* (undifferentiated mycelia) phenotypes were observed for strains 1787, T4 and J47a, respectively. (**B**) Response of *B. cinerea* B05.10 to different wavelengths of light. B05.10 was incubated during 10 d on solid CM in DD and LD employing light of different wavelengths. Transmitted light wavelengths were controlled using Petri dish chambers covered with Roscolux polyester filters. Thus, illumination with white light yields blue (BL), green (GL), yellow (YL) or red (RL) light, as it can be observed from the filter transmission spectra for the used filters (#381 Baldassari Blue, #389 Chroma Green, #312 Canary and #27 Med Red) indicated on top of each plate. (**C**) Linear growth rates of *B. cinerea* B05.10 grown under different light conditions. The strain was incubated in race tubes containing PDA medium under DD, LD and constant light (LL) conditions. After 7 d, race tubes were taken out and pictures were acquired from the top section of each tube. The vertical black lines indicate the growth fronts. (**D**) Quantification of linear growth rates observed in (C). The plot represents mean (± SEM) corresponding to the first 7 d of growth in race tubes. Letters indicate significant differences (p<0.001). (**E**) “Banding” phenotype of *B. cinerea* B05.10. The strain was incubated for 7 d in LD on solid CM supplemented with 0.02% SDS to reduce the daily growth rates (approx. 50% of radial growth on CM). The colony reached the edge of the Petri dish after 5 d of incubation. Each ring of conidia corresponds to one day (first ring was formed 2 d after inoculation).

A high level of genetic variation characterizes the species *B. cinerea*. Thus, different phenotypes with regard to the capability to produce secondary metabolites, the degree of virulence and the preferred mode of reproduction are observed in nature [40], [42], [58]–[64]. Paul [65] recognized on a first comparative study of *B. cinerea* three groups of isolates according to their morphological characteristics: *i)* those producing mostly conidia, *ii)* sclerotia, or *iii)* aerial (sterile) mycelia. Given the genetic/phenotypic variation observed in *B. cinerea*, a first step was to define a wild-type strain suitable for the systematic phenotypic and molecular study of light responses. B05.10 is an aggressive strain that was isolated in the 90′s from *V. vinifera* [39]. Due to its genetic stability and high rates of homologous recombination that allow the easy integration of different genetic constructs it has become, over the years, the standard recipient strain for genetic modifications in several laboratories [66]. B05.10 does undergo photomorphogenesis, i.e. the strain produces conidia in the light and sclerotia in the absence of this environmental cue (Figure 1A). In contrast, wild strains 1787, T4 and J47a present strikingly different photomorphogenic developmental programs. Irrespective of the lighting condition, strain 1787 and T4 are always forming sclerotia and conidia, respectively, while strain J47a is always producing undifferentiated mycelia of *“fluffy”* appearance (Figure 1A). Strains that lost the ability to form sclerotia are female-sterile. However, if they still form microconidia, they can act as male parent in sexual reproduction. The “*always conidia”* phenotype seems to be abundant in field populations although “*light-responsive”* strains (in terms of the presence of the mentioned reproductive structures) such as B05.10 are in a majority. The screening of 72 wild strains isolated from different host plants for the pursued differentiation program in light (LD) and continuous darkness (DD) revealed 57 strains (79%) forming conidia and sclerotia in a light-dependent fashion, one strain (1787) forming sclerotia in both culture conditions, 10 strains (14%) forming permanently conidia and four strains including J47a that failed to produce any reproductive structures (Figure 1A, and data not shown).

### Light is a key environmental signal influencing the mode of (asexual) reproduction in *B. cinerea* B05.10

To further confirm that B05.10 behaves like the *B. cinerea* strains used by Tan and colleagues to study light responses during the 70′s [67]–[74], we exposed cultures of B05.10 to different light wavelengths covering blue, green, yellow and red spectra. In agreement with the previous findings on *“light-responsive”* strains, B05.10 forms predominantly conidia in short-wave light (blue, green light) and sclerotia in long-wave light (yellow, red light) when applied in a 12:12 h light:dark photoperiod (Figure 1B). Nevertheless, exposure to blue light negatively affects conidia production while positively enhancing aerial (sterile) hyphae formation (Figure 1B, LD versus BL-D). This is in agreement with old reports describing the capacity of blue light to inhibit conidiation and to cause the “de-differentiation” of already developing conidiophore and sclerotial initials to sterile hyphae [69], [72], [75]. Therefore, while small dosages of white light suppress sclerotial development, it is blue but not the red fraction of the light spectra the one negatively affecting sclerotia formation (Figure 1B, YL-D and RL-D).

It is known from other fungi, such as *N. crassa* and *A. nidulans*, that the nutritional status influences the developmental programs in response to light [8], [76]. Therefore, we monitored conidiation/sclerotial development of B05.10 on different media (Figure S2A). They included minimal medium (MM), a defined complete medium (CM) as well as complex rich media containing plant components such as mashed potatoes and bean leaves (PDAB), vegetables (V8) and grape juice (GJ). In DD, B05.10 produced sclerotia when incubated on poor (MM) and rich media with one exception: the medium that was made from undiluted grape juice. Conversely, in LD, all employed media led to the production of conidia, while in the case of GJ, conidia were observed in both LD and DD. To test whether the osmotic potential of the medium – grape juice contains high amounts of sugars – does affect sclerotial development, we supplemented solid CM with sorbitol or NaCl. As observed in Figure S2B, higher osmolarities (1.4 M sorbitol, 0.7 M NaCl) suppress sclerotia formation, observing mostly conidia formation. It is worth mentioning that these reproductive structures are not observed in liquid cultures (data not shown). In aggregate, although light plays a key role in the control of asexual/sexual reproduction in B05.10, hyperosmotic conditions are able to overrule the effect of light. Taken together, we consider B05.10 a well-suited strain to study light perception and signaling in *B. cinerea* in which conidiation and sclerotia development can be easily scored as accessible phenotypic readouts.

### Light affects growth rates and pigment accumulation

Since light can exert a detrimental effect on organisms' physiology, we proceeded to compare growth rates and colony morphology of B05.10 cultures grown under excessive illumination (LL) or in the complete absence of this strong environmental cue (DD). Likewise, we examined other macroscopic phenotypic changes, such as the accumulation of pigments. In order to closely mimic environmental oscillations, in addition to LL and DD conditions, we also included 12:12 LD cycles. As *B. cinerea* grows very fast reaching the edge of the Petri dish within a short period of time, PDA-containing race tubes were used that allow monitoring growth for longer periods of time. As noted in Figure 1C and 1D, significant differences in the average daily linear growth rates were observed depending on the lightening conditions. Hence, light negatively affects growth rates yielding 61% (LL) and 82% (LD) of the recorded daily growth rate observed in DD. This result indicates that light especially when applied in excess (LL) represents a stress factor for *B. cinerea*. Interestingly, the growth retardation in LL was accompanied by the accumulation of a dark pigment. During incubation in LD conditions, B05.10 forms a regular “banding” pattern, hence, grayish “bands” due to conidiation in the light are followed by white “bands” due to the absence of conidiation in the dark (Figure 1C). This growth pattern is also visible during LD incubation in Petri dishes where daily growth rates have been artificially decreased by the addition of SDS (Figure 1E), and does not appear during incubation in LL (Figure 1C) or in strains (e.g. T4) exhibiting the “*always conidia”* phenotype (Figure 1A).

### *B. cinerea* possesses several photoreceptors that could regulate differentiation, an it responds to light at the transcriptional level

**Figure 2:**
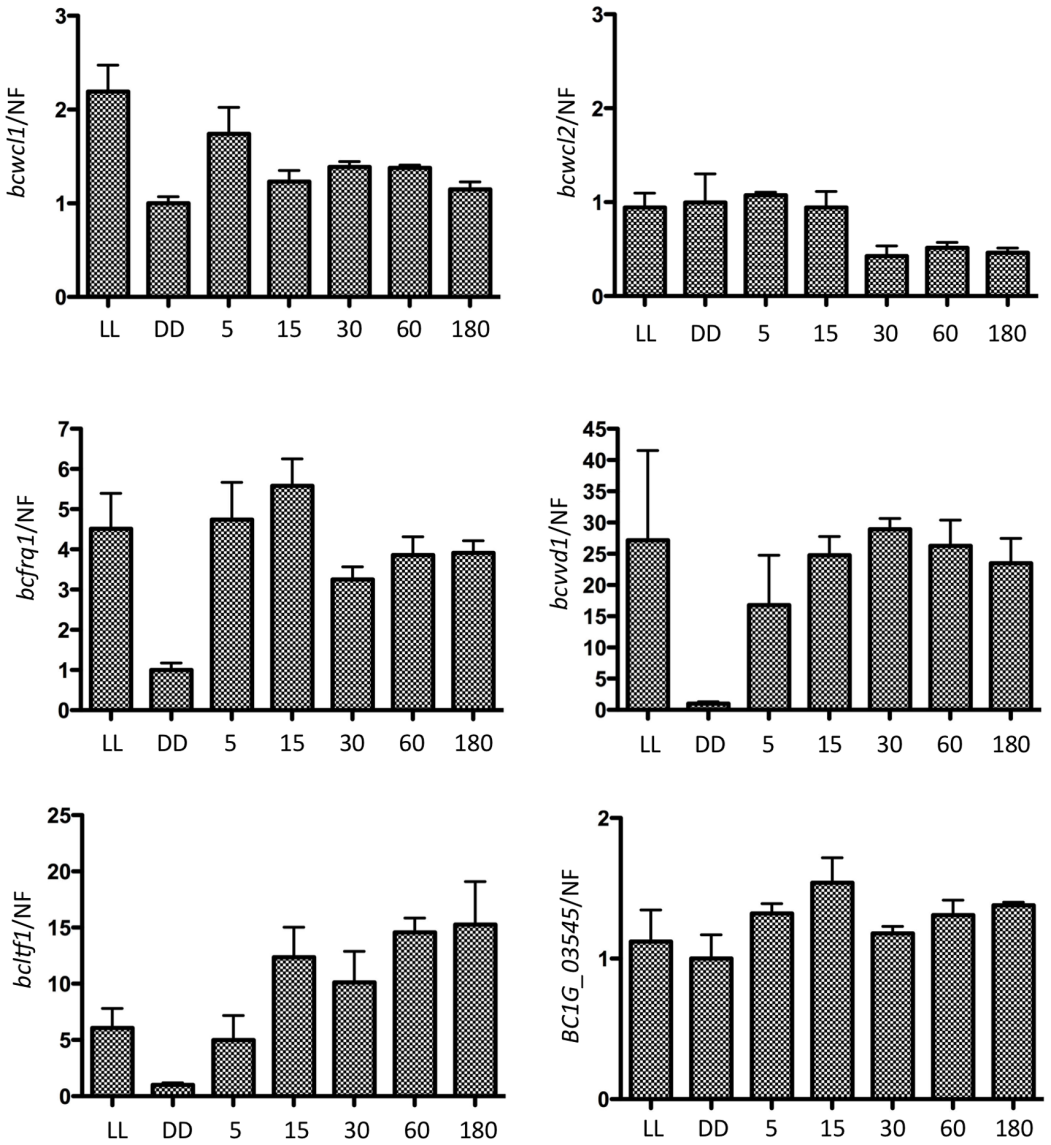
White light leads to a fast increase in transcript levels of selected genes in *B. cinerea*. Gene expression was analyzed by RT-qPCR as described in methods. Values are referred to DD conditions (control  = 1). Bars represent mean values ± SEM. A normalization factor (NF) was calculated for gene expression normalization (see methods). Values were calculated from two biological replicates with two technical replicates each. Primer pairs employed for RT-qPCR amplification are indicated in Supplementary Table S1.

In agreement with different light wavelengths modulating morphogenesis in *B. cinerea* [37], [38], genes encoding for orthologs implicated in light perception and transduction in *N. crassa* [4] can be identified in its genome [3], [77], including a VIVID-like protein as potential blue light receptor and an ortholog of the WC-1 blue light receptor/TF. In addition, *B. cinerea* possesses genes encoding cryptochromes as potential UV/blue light sensors (*bccry1*, *bccry2*), opsins (*bop1*, *bop2*) and red light-sensing phytochromes (*bcphy1*, *bcphy2*, *bcphy3*) ([3], [77]). Moreover, genes encoding orthologs of the circadian clock component FRQ (*bcfrq1*), and WC-2 (*bcwcl2*), as well as light-responsive TFs can be identified in the genome database. These include SUB-1 (*bcltf1*), SAH-1 (*bcsah1*; *B. cinerea* light transcription factor 2 *bcltf2*), VAD-3 (*bcvad3*) and CSP-1 (*bccsp1; bcltf3*) [78] all involved in asexual/sexual developmental processes *in N. crassa*. Thus, in order to assess the transcriptional impact of light on key selected genes, young undifferentiated mycelium was chosen to monitor the expression of some putative light-responsive genes. This type of tissue, when grown in the absence of light, is fully developmentally competent and able to bear conidia or sclerotia depending on the following illumination event. For this, strain B05.10 was cultivated for 2 d in DD on solid medium (PDA) covered with a cellophane overlay (see methods). Then, DD-grown cultures were further kept in the dark or exposed to white light pulses for periods of 5, 15, 30, 60 or 180 min. RNA was subjected to RT-qPCR, evaluating the transcript levels of *bcwcl1*, *bcwcl2*, *bcfrq1*, *bcvvd1* and *bcltf1* (Figure 2). Short light pulses (5 min) were sufficient to induce the expression of *bcfrq1* (5-fold), *bcvvd1* (15-fold) and *bcltf1* (5-fold), though transcript abundance of the latter gene further increased by prolonged exposure to light (approx. 15-fold after 180 min). Expression of *bcwcl1* and *bcwcl2* was not significantly affected by short-term light treatments, but a two-fold increment of transcript levels was observed for *bcwcl1* during incubation in LL (p<0.05, t-test). Likewise, transcript levels of the other genes under analysis were elevated in LL compared to DD. As a negative control, we chose the ortholog of the gene encoding for the *N. crassa* ACON-3/*A. nidulans* Med*A* (BC1G_03545), a transcriptional regulator that controls conidiation in *N. crassa* [79] and that is not light-induced. As its ortholog in the latter organism, expression of BC1G_03545 is not responsive to light (Figure 2). Additional RT-qPCR experiments (see below) showed a light-dependent increase in the mRNA levels of further genes encoding photoreceptors and TFs. In aggregate, these findings demonstrate that *B. cinerea* responds to light at the transcriptional level.

### Unraveling the functions of the *white collar* transcription factors in *B. cinerea*: BcWCL1 mediates transcriptional responses to white light

With the premise that light responses and associated molecular components exhibit significant conservation among filamentous fungi, we focused on the *white collar*-like TFs. These proteins in *N. crassa* form a complex that can directly respond to a light stimulus and activate gene expression, a mechanism that has also been confirmed in *A. nidulans* [1].

The ORF of *bcwcl1* (*white collar 1*-like) comprises 3,765 bp, is interrupted by a single intron of 351 bp located towards the 3′-end of the gene and encodes a protein of 1,137 aa. Like its counterparts in other fungi, BcWCL1 contains a LOV domain, two PAS domains, a putative NLS (^922^RKKRKRRK^929^) and a GATA-type zinc-finger DNA-binding domain at the C terminus (Figure S3). BlastP analyses revealed overall amino acid identities of BcWCL1 to proteins of *S. sclerotiorum* (SWC1; 1,146 aa), *N. crassa* (WC-1; 1,167 aa) and *A. nidulans* (LreA; 837 aa) of 75, 46 and 41%, respectively. The 509-aa long BcWCL2 is encoded by an ORF of 1,723 bp with two introns (128 and 65 bp), and contains a single PAS domain, a putative NLS (^422^KKKK^445^) and a GATA-type DNA-binding domain. In contrast to BcWCL1, BcWCL2 shares higher and lower degrees of similarity with the orthologs proteins from *N. crassa* (53% aa identity with WC-2) and *A. nidulans* (35% aa identity with LreB), respectively.

Recent BImolecular Fluorescence Complementation (BIFC) assays demonstrated that BcWCL1 and BcWCL2 proteins can interact in the nuclei of vegetative hyphae of *B. cinerea* [55] confirming the existence of a BcWCL1/BcWCL2 complex (WCC) in this organism. To assess whether this complex is capable of mediating transcriptional responses to light and also to affect differentiation in *B. cinerea*, mutants with altered WCC activities were functionally characterized. For that, we first deleted *bcwcl1* from the genome in order to prevent WCC signaling. Based on the central role of WC-1 as the light-sensing moiety of the WCC, *bcwcl1* deletion mutants are expected to be devoid of WCC blue-light responses and for functional purposes are generally considered WCC deletion mutants [10].

**Figure 3:**
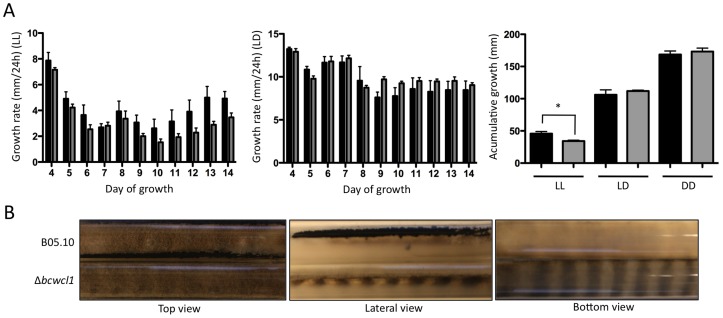
Light negatively affects *B. cinerea* linear growth rates. (**A**) Linear growth rates of wild-type (black bars) and Δ*bcwcl1* (grey bars) strains were measured in race tubes assays. For LL and LD conditions, growth rates were determined daily (left and central panel). The accumulative growth (right panel) was determined for LL, LD and DD conditions after 14 d of incubation. Each bar represents the mean ± SEM of three independent Δ*bcwcl1* mutants (four technical replicates each). Statistical differences (p<0.05) are indicated with asterisks. (**B**) Phenotypic characterization of the Δ*bcwcl1* mutant (clone 1) grown for 7 d in race tubes under LD conditions.

At a first glance, when wild-type and Δ*bcwcl1* strains were cultivated in Petri dishes, both strains exhibited comparable growth rates in all tested light conditions (data not shown). However, long-term growth assays performed in race tubes demonstrated that Δ*bcwcl1* mutants exhibited wild-type-like growth rates in DD, but reduced daily growth rates under LL conditions when compared to the wild-type strain. As shown in Figure 3, a significantly reduced accumulative growth after 14 d of incubation (75% of the growth determined for the wild-type in LL) was observed for Δ*bcwcl1*. Importantly, Δ*bcwcl1* as well as the wild-type displayed a sustained and marked reduction in their growth rates in LL (20% and 27% of the growth observed in DD, respectively). However, light applied as a LD regime, decreased growth rates of both strains in a similar manner (65 and 63% of the growth observed in DD, respectively). It is worth mentioning that while the DD data shown in Figure 1 was acquired daily using low-intensity red-safety lights, the DD cultures from Figure 3 were never subjected to any light source until the end of the experiment, when the growth front was measured. Notably, the characteristic regular “banding” pattern shown in Figure 1 was even more pronounced in the Δ*bcwcl1* strain. Bottom or lateral views of the race tubes allow an evident visualization of this phenotype (Figure 3B). Similar “banding patterns” were observed during incubation in white and red light (long-wave light; Figure S4).

**Figure 4:**
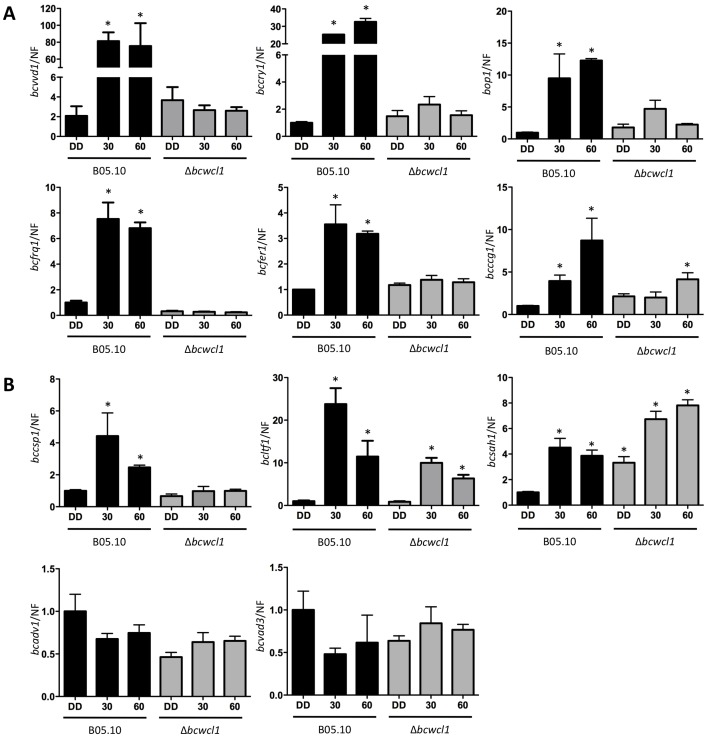
The GATA-type TF BcWCL1 mediates some – but not all – light-dependent transcriptional changes in *B. cinerea*. Transcriptional responses to (white) light pulses (30 and 60 min) for several light-responsive genes non-coding (**A**) and coding for TFs (**B**) are shown for wild-type B05.10 and the Δ*bcwcl1* mutant. Values are referred to B05.10 grown in DD (control). Bars represent mean ± SEM. A normalization factor (NF) was calculated to normalize gene expression data (see methods). Values were calculated from three biological replicates with two technical replicates.

To assess the impact of WCC on the previously described transcriptional responses to white light, wild-type B05.10 and the Δ*bcwcl1* mutant were incubated in the dark and then exposed to light for 30 and 60 min (Figure 4). Expression levels of all analyzed genes encoding for predicted photoreceptors (*bcvvd1*, *bccry1*, *bop1*; Figure 4A) were strongly induced by light in the wild-type while no significant differences were observed in the Δ*bcwcl1* mutant, although a transient (but not statistically significant) induction was observed for the latter photoreceptor-encoding gene in the Δ*bcwcl1* genetic background. Known *N. crassa* WCC targets such as *bcfrq1* and *bcfer1* (the latter gene encoding for ferrochelatase, which has been proposed as an ancient target of photoregulation in the fungal kingdom [80]), as well as *bcvvd1,* were induced by light only in the wild-type strain. *Bcccg1*, which encodes for the ortholog of the *clock-control gene 1* of *N. crassa,* showed light-inducibility in both wild-type and Δ*bcwcl1* strains. On the other hand, the genes encoding for putative TFs that were analyzed, showed contrasting results. While *bccsp1* was significantly induced upon light simulation only in the wild-type strain, both *bcltf1* and *bcsah1* showed a clear light-dependent induction in both strains (e.g. 24- and 10-fold for *bcltf1* in the wild-type and Δ*bcwcl1*, respectively). Moreover, putative light-responsive TFs encoding genes *bcadv1* and *bcvad3* were not light-induced. In aggregate, these results suggest that in addition to the WCC, other molecular systems have an important role in controlling the light-dependent expression of some light-inducible genes. Thus, although BcWCL1 plays a central role in mediating light perception and activating gene expression in *B. cinerea*, importantly, and in contrast to what has been shown in *N. crassa*, transcriptional responses to light are overtly detected in this mutant.

### The BcWCL1/BcWCL2 complex regulates light-dependent differentiation

**Figure 5:**
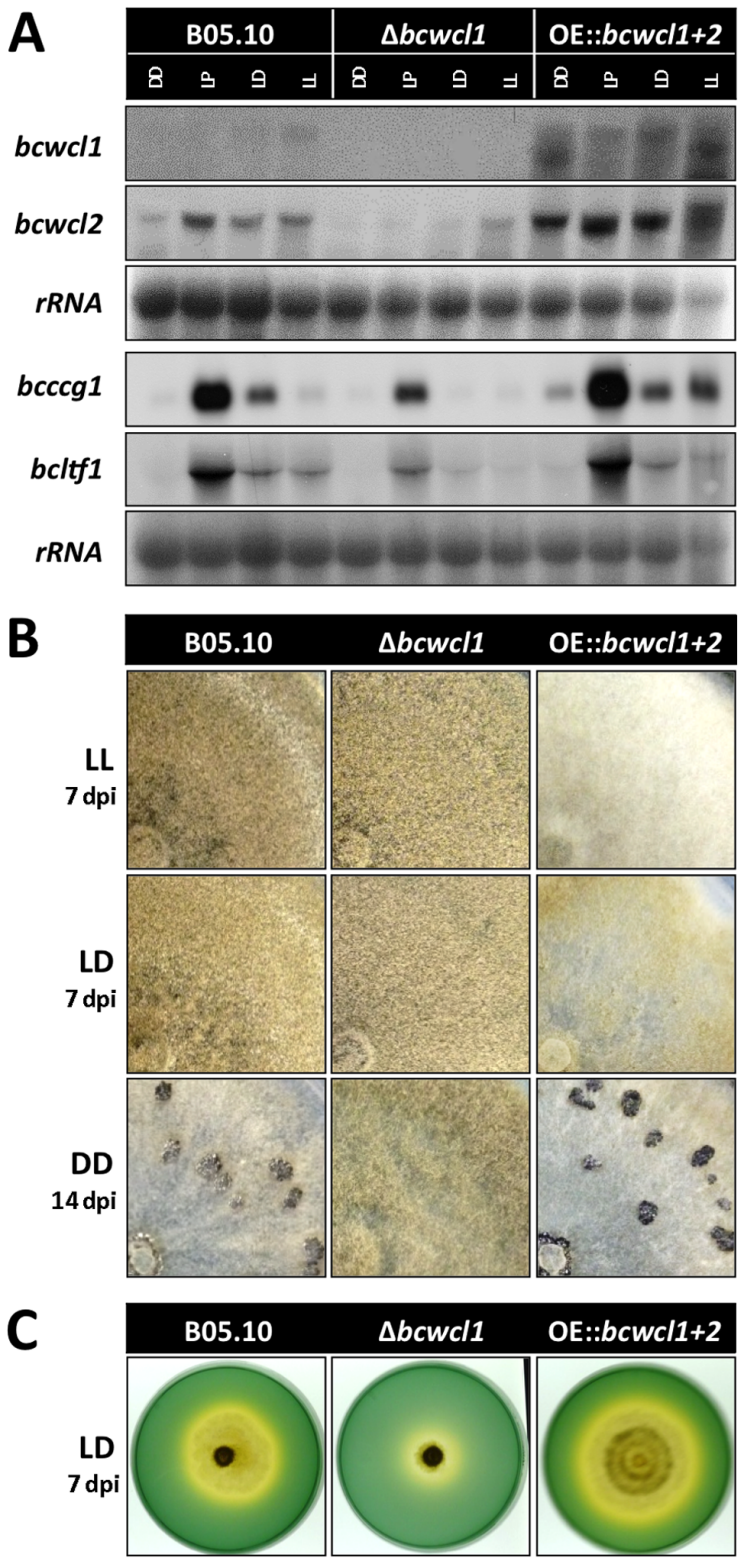
Deletion of *bcwcl1* as well as the simultaneous overexpression of *bcwcl1* and *bcwcl2* affects light-dependent differentiation. (**A**) Overexpression of *bcwcl1* and *bcwcl2* affects the expression of light-induced genes. Strains (indicated at the top of the figure) were incubated during 48 h on solid CM covered with cellophane in LL, LD (after 6 h in the lights-on period) and DD conditions, or exposed for 1 h to light (LP) after 48 h in DD. rRNA is shown as loading control. (**B**) Deletion of *bcwcl1* and overexpression of *bcwcl1* and *bcwcl2* affects light-dependent differentiation. Strains (indicated at the top of the figure) were incubated on solid CM medium in LL, LD and DD culture conditions. (**C**) *Bcwcl1* mutants are impaired in their ability to acidify the culture medium. Acidification due to oxalic acid secretion was monitored on solid CM, pH 7.5, supplemented with bromothymolblue employed as pH indicator. The change in color from green to yellow denotes acidification (pH<6.0).

To further characterize the impact of light and the WCC on light-dependent differentiation programs in *B. cinerea*, we took advantage of previously generated strains expressing *bcwcl1* and *bcwcl2* under control of constitutive promoters [55]. As shown in Figure 5A, we confirmed that under all culture conditions tested both *bcwcl1* and *bcwcl2* were higher expressed in the respective mutant (referred hereafter as OE::*bcwcl1+bcwcl2*) when compared to the wild-type and Δ*bcwcl1* strains. Interestingly, the overexpression of the WCC only exerts minor effects on the expression of *bcltf1* (Figure 5A), which in conjunction with the results depicted in Figure 4 suggest that the WCC is not the only regulator of *bcltf1* expression.

Notably, and highlighting the role of the WCC on development, the OE::*bcwcl1+bcwcl2* displayed, particularly in LL, and to a lesser degree in LD, increased formation of aerial hyphae associated with reduced conidiation, yielding colonies with a *fluffy* appearance (Figure 5B). In contrast, growth characteristics of the OE::*bcwcl1+bcwcl2* mutant in DD resembled those of the wild-type. Importantly, strains overexpressing only BcWCL1, or BcWCL2, exhibited a wild-type-like phenotype (data not shown). Nevertheless, and based on the slight increase in the mRNA levels of *bcccg1* in DD, we cannot discard an enhanced WCC activity in the absence of light (Figure 5A). Furthermore, Δ*bcwcl1* and OE::*bcwcl1+bcwcl2* exhibited different capacities to grow under alkaline conditions (pH 8) indicating that the WCC may be involved in the regulation of oxalic acid production (Figure 5C).

In contrast to the OE::*bcwcl1+bcwcl2* mutant that developed either sterile hyphae in LL or sclerotia in DD, the deletion of *bcwcl1* resulted in mutants that produced conidia under all light conditions (Figure 5B). Remarkably, conidiation of Δ*bcwcl1* colonies started earlier than wild-type colonies. Responses of Δ*bcwcl1* to the different light conditions were still detected, as conidiation in the presence of light initiated earlier than in its absence (Figure S5A). As described for the race tubes assays, the characteristic “banding pattern” in response to LD conditions was even more pronounced than that of the wild-type as observed also in Petri dishes (Figure S5B).

Taken together, the fact that the deletion of *bcwcl1* leads to precocious and persistent conidiation while the overexpression of the TFs prevents conidial development suggest that the BcWCL1/BcWCL2 complex mediates the suppression of conidiation by forcing the proliferation of aerial hyphae in response to (blue) light.

### The *bcwcl1* gene complements the *bcwcl1* deletion mutant in *B. cinerea*

Three independent deletion mutants for *bcwcl1* (Δ*bcwcl1*-1 to −3) having single integration of the replacement cassettes (Figure S1, Table S2) were generated and confirmed to exhibit the same phenotype. Strain Δ*bcwcl1-*1 was arbitrary chosen as recipient for genetic complementation to fully establish that the deletion of *bcwcl1* explains the observed light-dependent phenotypes. As shown in Figure S6A, *bcwcl1* was targeted to the *bcniaD* locus by homologous recombination yielding Δ*bcwcl1+bcwcl1*. As expected, in the absence of light, strain B05.10 formed sclerotia in contrast to Δ*bcwcl1* which persisted in conidiation, while the expression of *bcwcl1* in the Δ*bcwcl1* background restored sclerotia formation (Figure S6B). In addition, light-inducibility in the complemented strain was analyzed by RT-qPCR. As observed in Figure S6C, light induction of both *bcfrq1* and *bcvvd1* were recovered in Δ*bcwcl1+bcwcl1* to similar levels when compared to strain B05.10. In aggregate, these results confirm that the hyper-conidiation phenotype accompanied by the loss of sclerotial development and the absence of light-dependent transcriptional responses observed for the *bcwcl1* mutant are due to the deletion of *bcwcl1*.

### The BcWCL1/BcWCL2 complex is involved in oxidative stress response

**Figure 6:**
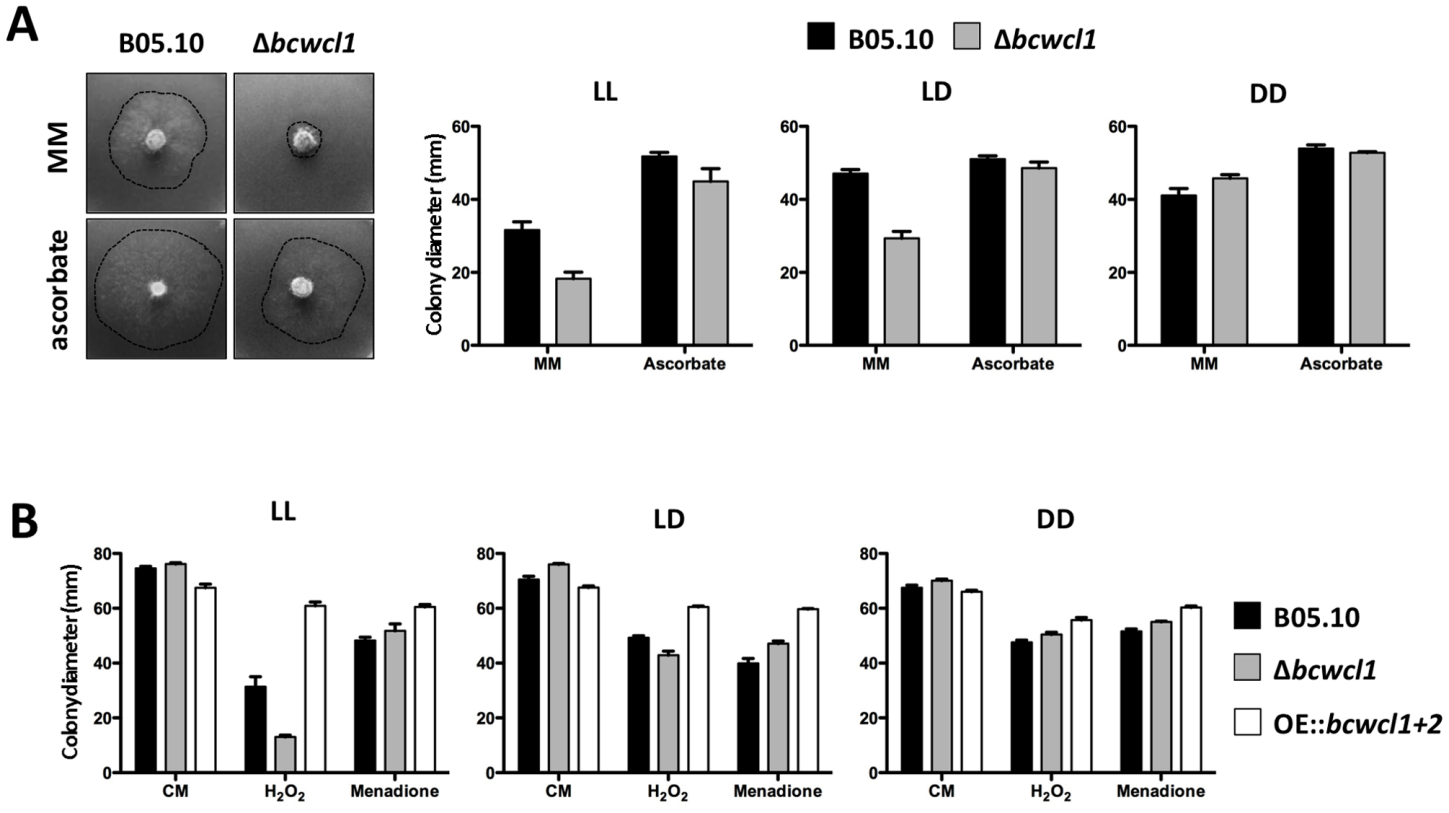
Deletion of *bcwcl1* and overexpression of the WCC affect the response to oxidative stress. **(A)** Ascorbate increases growth rates of wild-type B05.10 (black bars) and Δ*bcwcl1* (grey bars) strains in the presence of light (LD, LL). Both strains were incubated for 3 d on MM (minimal medium) supplemented with 5 g/l ascorbate. Mean values ± SEM were calculated from five colonies per strain in each condition. (**B**) Overexpression of *bcwcl1* and *bcwcl2* decreases the sensitivity to oxidative stress. Strains were grown for 3 d under LL, LD or DD conditions on solid CM in the absence of stressors agents (control) and in the presence of 7.5 mM H_2_O_2_ or 300 µM menadione as indicated in the figure. Mean values ± SEM were calculated from five colonies per strain grown in each condition.

Light when applied in excess (LL) exerts detrimental effects on growth rates of *B. cinerea* (Figure 1 and 3) which could be due, in part, to perturbation in the homeostasis of cellular ROS (Reactive Oxygen Species) levels. To evaluate this possibility, we exposed B05.10 and Δ*bcwcl1* strains to LL, LD and DD culture conditions with and without an antioxidant (5 g/l ascorbic acid), thus increasing the antioxidant potential of the cell on minimal medium (MM) (Figure 6A). Light sensitivity (measured as colony diameter) of Δ*bcwcl1* was already detectable in LD and became more pronounced in LL (63% and 39% of the growth observed in DD, respectively), whereas the wild-type exhibited comparable growth rates in DD and LD and slightly reduced growth rates in LL (75% of the growth observed in DD). Notably, the addition of ascorbic acid restored the light-dependent reduction in growth observed for Δ*bcwcl1* to wild-type levels, and both B05.10 and Δ*bcwcl1* strains recovered DD-like growth levels in the presence of light (LL) and ascorbate, indicating that light can generate oxidative stress and that the action of the WCC is needed to cope with this strong environmental variable.

As a further proof of the connection between light sensitivity and ROS, we exposed wild-type, Δ*bcwcl1* and OE::*bcwcl1+bcwcl2* strains to oxidative stress caused by hydrogen peroxide (H_2_O_2_) or menadione, under different illumination conditions (Figure 6B). We hypothesized that if the WCC is involved in oxidative stress response, in the presence of H_2_O_2_ and light (LL), the absence of the WCC would lead to an even more drastic (negative) impact on growth, due to the enhanced effect of combining both stressor agents, while on the other hand, the overexpression of the mentioned complex would result in a more resistant phenotype. Similar growth rates were observed for all strains when incubated in DD, while the presence of light increased the toxic effect of H_2_O_2_ but not that of menadione. Hence, the wild-type strain was impaired in coping with H_2_O_2_ in LL exhibiting a 58% reduction in growth rate in comparison with the culture grown in the absence of the stressor agent. As expected, the Δ*bcwcl1* mutants displayed an increased sensitivity to H_2_O_2_ in LL, registering only a 26% of the growth rate achieved in DD in the presence of H_2_O_2_. In contrast, the OE::*bcwcl1+bcwcl2* mutant was insensitive to the combination of light and H_2_O_2_ (approx. 110% of the growth observed in DD) and consequently more resistant to H_2_O_2_ in the presence of light than the wild-type (194% of the growth observed for the wild-type).

### BcWCL1 is required for full virulence in the presence of light

**Figure 7:**
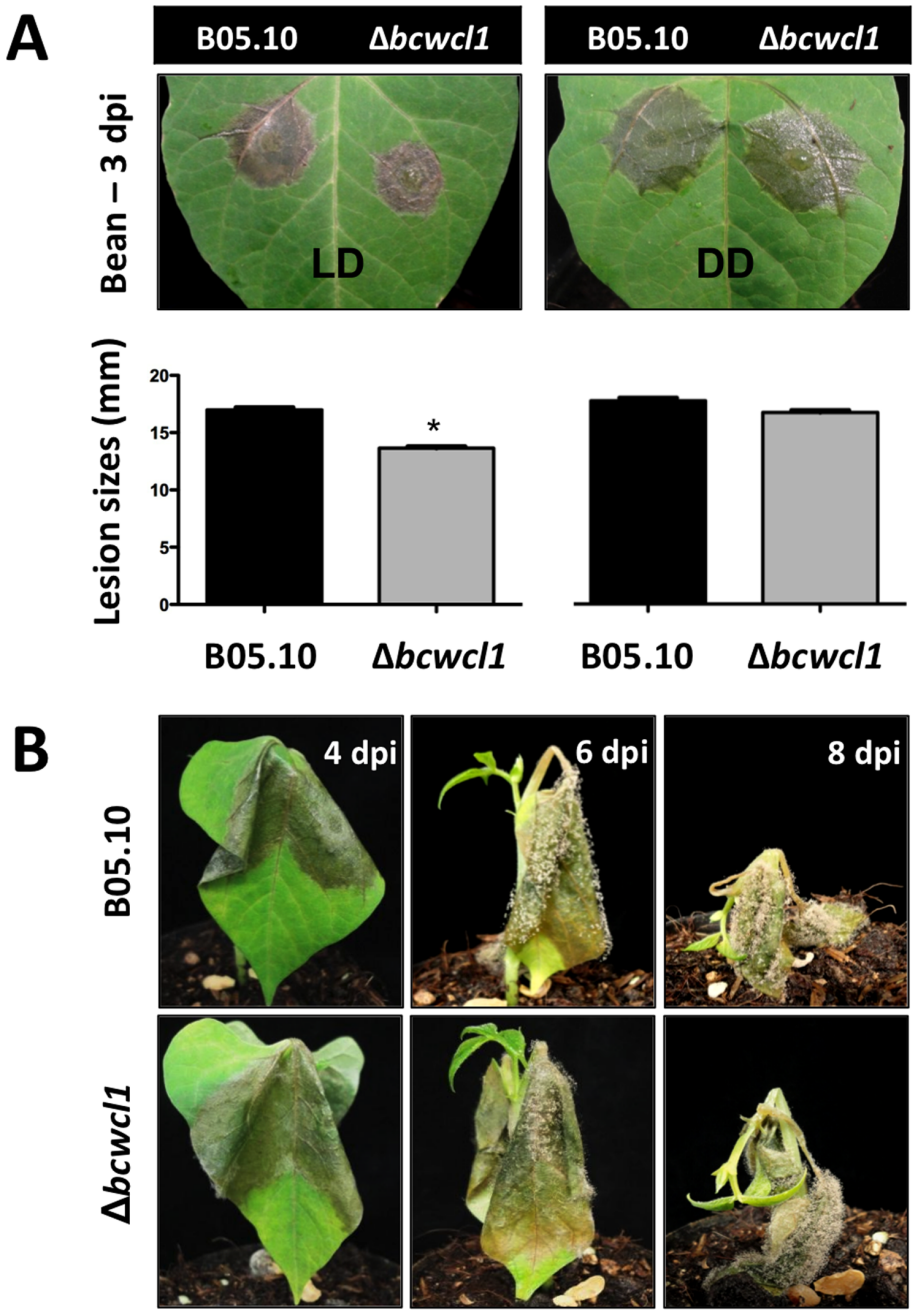
Virulence of Δ*bcwcl1* mutants is impaired in a light-dependent fashion. (**A**) Lesion spreading of Δ*bcwcl1* mutants is slightly affected in LD but not in DD. Primary leaves of living plants (*P. vulgaris*) were inoculated with conidial suspensions and incubated for 3 d in humid conditions under LD or DD conditions. Mean values ± SEM of lesion diameters were calculated from 22 lesions per strain and light condition, with two measurements per lesion. Statistical differences (p<0.05) are indicated with asterisks. (**B**) Soft rot formation and conidiation are not affected by the deletion of *bcwcl1*. No significant differences were observed when inoculated plants were incubated in LD or DD after 4 dpi and over. Plants incubated in LD are shown.

To gain insight into the relevance of light and the WCC in the *B. cinerea*-plant interaction, we assayed virulence of the *bcwcl1* deletion mutant on French bean (*P. vulgaris*) and *A. thaliana* Col-0 plants representing highly and moderately susceptible hosts of *B. cinerea*. Reduced lesion sizes were observed on *P. vulgaris* plants that were incubated for 3 d in LD but not for those incubated in DD (Figure 7A). However, from 4 dpi and over, no further differences between the wild-type and the *bcwcl1* deletion mutant were detected as both strains finally proceeded to colonize the plant tissue, ending in soft rot and conidiation (Figure 7B).

**Figure 8:**
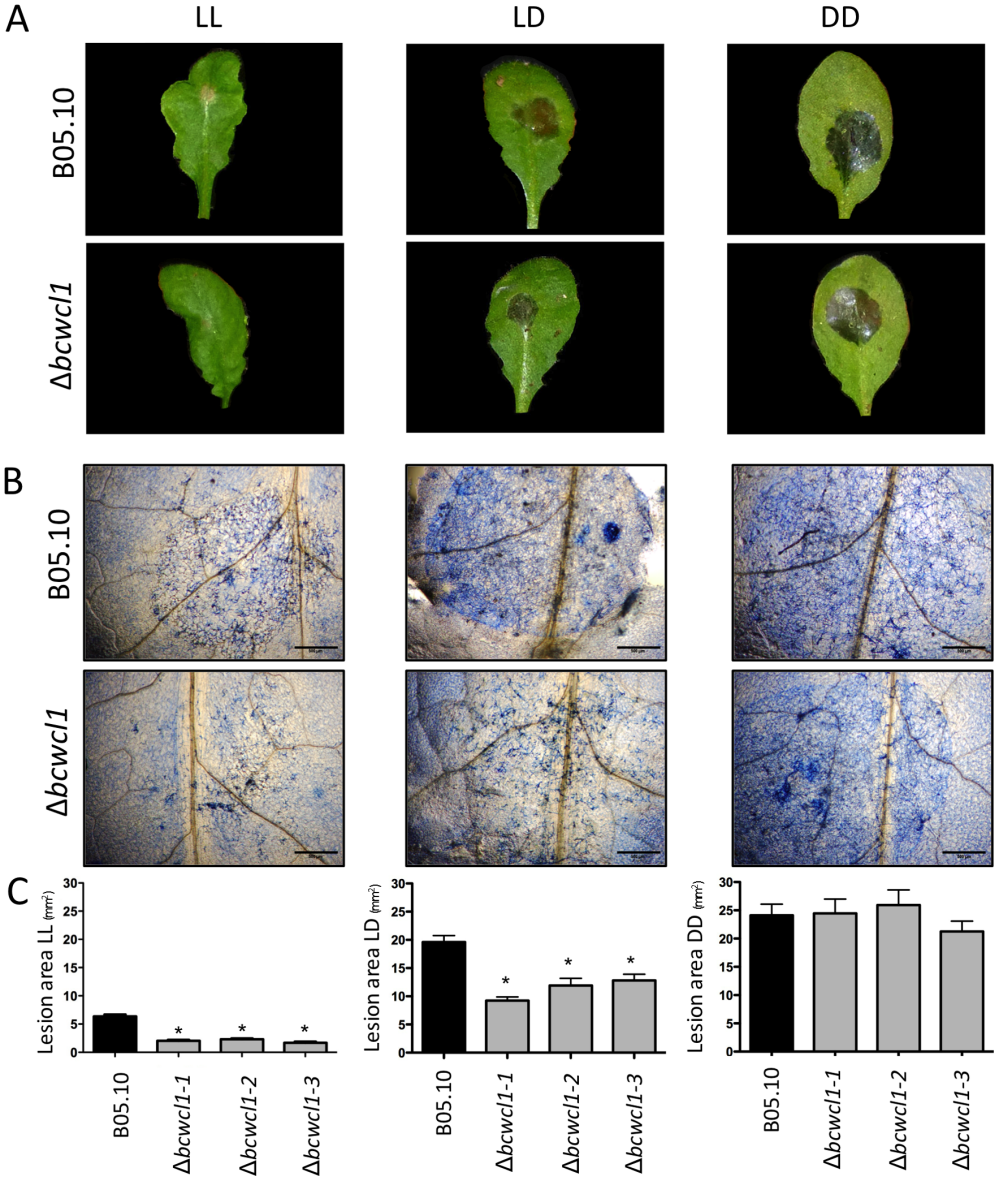
BcWCL1 is required to achieve full virulence in the presence of light. (**A**) Conidia (2x10^5^/ml, 7 ml) from the wild-type B05.10 strain (top) and a representative Δ*bcwcl1* mutant (bottom) were inoculated on approximately 1-month-old *A. thaliana* (Col 0) plants, grown at 20°C under LD (12:12 h) conditions. After spore inoculation, plants were grown for 3 days under LL, LD or DD conditions. Pictures were acquired after 3 dpi. Representative leaves for each culture condition are shown. (**B**) Trypan blue staining indicates fungal growth in plant tissues at 3 dpi (black scale bars represent 500 µm). (**C**) Quantification of lesion areas, obtained from at least four independent assays for each Δ*bcwcl1* mutant. Bars represent mean values ± SEM. Significant differences in comparison with the lesion areas observed for B05.10 are indicated with asterisks (p<0.05).

The impact of light on the plant-pathogen interaction was more precisely analyzed using *A. thaliana* as a host. First, plants were normally grown using 12:12 h light:dark photoperiods, and thereafter incubated in LL, LD and DD. Remarkably, light conditions already severely affected the infection of plants by the wild-type strain. Accordingly, and in comparison with the DD culture condition, 65% (LL) and 19% (LD) reductions in the lesion areas on *A. thaliana* leaves were observed for the wild-type strain (Figure 8A and C). Further reductions of lesion areas were observed for the Δ*bcwcl1* mutant in a light-dependent manner (in comparison with the DD culture condition, 85% (LL) and 53% (LD) reductions). Reduced proliferation of fungal material on the host was furthermore confirmed by trypan blue staining (Figure 8B). Since plant responses to abiotic and biotic stress conditions are characterized by an oxidative burst, and Δ*bcwcl1* mutants are hypersensitive to H_2_O_2_ under LL conditions, we evaluated the accumulation of H_2_O_2_ in infected plant tissues by using 3,3′-diaminobenzidine (DAB). However, no differences between wild-type- and mutant-infected plant tissues in any light condition were observed (Figure S7).

## Discussion

For over a century, it has been known that light represents a key environmental cue for the plant pathogenic fungus *B. cinerea*. Early studies underlined light importance on tropic responses of conidiophores, conidial germ tubes and fruiting bodies [81]–[85], while others described light impact on fungal morphogenesis, promoting conidiation and suppressing sclerotial development [86], [87]. Nevertheless, to date no molecular approaches have been undertaken to address this phenomenon. Herein, we have provided evidence that *B. cinerea* responds to light stimuli, implicating the participation of a TF/blue-light photoreceptor complex in this process.

Understanding the effect of light on *B. cinerea*, particularly in terms of influencing its developmental program, has not been trivial. Confusing observations have been generated by the isolation and study of different *B. cinerea* strains exhibiting altered or no responses to light. Regardless of the lighting conditions, some strains lead to persistent conidiation, sclerotia formation or even to the absence of any reproductive structures [59], [65]. Moreover, studies undertaken in the 1970′s demonstrated that the fungus does respond to different light wavelengths, spanning those from near-UV to far-red light. Thus, Tan [70] postulated a “Two-receptor-model” in which near-UV/blue-reversible and red/far-red-reversible photoreceptors are closely interacting to control conidiation. During the past decades, photoreceptors have been identified and functionally characterized in *N. crassa* and *A. nidulans*, the best-characterized fungal photobiology models described so far. The analysis of these systems has revealed similarities as receptors for UV, blue light, red and far-red light sensors have been identified (reviewed in [4], [88]). Nevertheless, although several fungi share the same *in silico* repertoire of photoreceptors, functional differences have been observed: while the red-light signaling components physically interact with the blue-light sensing LreA/LreB complex in *A. nidulans* [22], no red-light responses have been detected in *N. crassa* [10], [25]. In this regard, among the orthologs of photoreceptor-encoding genes identified in the genome of *B. cinerea* [3] in contrast to *A. nidulans* (FphA) and *N. crassa* (PHY-1, PHY-2), three gene models encoding for putative phytochromes have been identified [43] suggesting an active role of red/far-red light in its lifecycle. Whether they interact with the WCC as it has been demonstrated in *A. nidulans*, remains as an open question.

Considering the possibility that light perception may modulate the properties of being a successful pathogen either by affecting the infection process or the overall fitness by impairing spread of disease (via conidia), survival or sexual recombination (via sclerotia and apothecia), we initiated molecular studies on conserved components of fungal light perception in *B. cinerea*. The *white collar* complex is formed by two GATA-type TFs, in which the WC-1 blue light-sensing domain exerts a central and conserved role in fungal light regulation, as shown in the ascomycetes *N. crassa* WC-1/WC-2 [9], [89], *A. nidulans* LreA/LreB [22], *T. atroviride* BLR-1/BLR-2 [90], zygomycetes (*P. blakesleeanus* MadA/MadB [91], [92]) and basidiomycetes (*C. neoformans* BCW1/BCW2 [28]). As shown here, *B. cinerea* encodes for orthologs of the transcription factors WC-1 and WC-2, which exhibit characteristic key conserved domains.

*B. cinerea* responds to white light at the transcriptional level as seen by the increase in expression levels for genes encoding photoreceptors and TFs. Remarkably, although in *N. crassa* the WCC is responsible for the increase in transcript abundances of almost all analyzed light-responsive genes, in *B. cinerea* the orthologs of several important genes remained light-responsive in the absence of *bcwcl1*, indicating that the function of the WCC to drive gene expression in response to light is only partially conserved in *B. cinerea*. Shared targets of the WCC in *B. cinerea* and *N. crassa* include genes encoding for heme biosynthesis, proteins likely to be part of a yet uncharacterized circadian clock and putative blue light photoreceptors including *bcvvd1* and *bccry1*. Future work will assess the role of BcVVD1 in photoadaptation and the participation of BcCRY1 in mediating light responses and its potential role in DNA repair mechanisms. Importantly, it has been described that upon light stimulation, the WCC recognizes the promoter of over 20 genes encoding for TFs in *N. crassa*, leading to a hierarchical transcriptional cascade. When we analyzed the behavior of some of the orthologs in *B. cinerea*, we observed interesting differences. While light induction of *csp-1*, *sub-1* and *sah-1* directly depends on the WCC in *N. crassa*, only *bccsp1* expression looses light responsiveness in the Δ*bcwcl1* background. In contrast, *bcltf1* (*sub-1* in *N. crassa*) and *bcsah1* were still expressed in a light-dependent fashion in this mutant. Interestingly, for the latter TF-encoding gene, expression levels are even higher in the absence of BcWCL1. In contrast, *bcvad3* or *bcadv1* levels are not increased by light, although the expression of the *N. crassa* orthologs has been shown to respond to this stimulus [10]. In aggregate, these results suggest that first-tier targets of WCC in *N. crassa* are not totally conserved in *B. cinerea*, opening up interesting questions related to the evolution of transcriptional networks and underlying physiology changes that are triggered upon light stimulation in this plant pathogen.

Divergence of downstream signaling events may reflect that light differentially affects the lifestyles of different fungi, even those of closely relates species. For example, light strongly favors conidiation in *A. nidulans* [93], minimally affects this process in *A. fumigatus* [24], while it represses this process in *A. oryzae* [94]. Also, interesting differences related to sexual and sclerotial development are observed in response to light and its absence, respectively, in various Aspergilli [95]. In the genus *Trichoderma*, White Collar orthologs have also been shown to regulate light responses, showing strong evidence for light-repressed genes [96]. Similarly, while several genes are positively regulated by light in *C. neoformans* [80], other processes as mating are negatively affected [28].

As shown here, light is able to regulate the mode of reproduction in *B. cinerea* being an “absolute” signal as either conidia or sclerotia are produced. This differs from what has been described in *N. crassa* in which the formation of protoperithecia demands nutrient limitation and light only plays a promoting role [8], while in *A. nidulans* light and other stresses just alter the ratio of formed conidia and cleistothecia [1]. Importantly, the effect of light on differentiation can be decomposed on the individual or combined effect of particular wavelengths. Thus, *N. crassa* as well as other species of the Sordariomycetes have been reported to exclusively respond to blue light while *A. nidulans* belonging to the Eurotiomycetes additionally senses and react to near-UV and red light [21], [22], [97] as *B. cinerea*, a member of the Leotiomycetes. Therefore, the light signaling machinery in *B. cinerea* appears more complex than that of *N. crassa.*

Despite the fact that several filamentous fungi employ light as an environmental cue that provides information on the whereabouts, they have to cope like most organisms with the detrimental effects of light. Considering that *B. cinerea* is adapted to natural light conditions, the wild strain B05.10 exhibits comparable growth rates in LD and DD under laboratory conditions. However, excessive illumination (LL) significantly impairs growth of the wild-type strain and even more that of the Δ*bcwcl1* mutants. The negative effect of light can be enhanced and reversed by applying additional oxidative stress (H_2_O_2_) and antioxidants, respectively, indicating that light causes oxidative stress and that the WCC is mediating the adaptation to this condition. Thus, it is possible to hypothesize that among the blue light/WCC target genes are those encoding enzymes involved in ROS detoxification and/or DNA repair.

Changes in the intracellular ROS levels are known to trigger differentiation in *B. cinerea*. The NADPH oxidase (NOX) complex, as a producer of superoxide radicals, is required for sclerotial development and germling fusions via conidial anastomosis tubes [98], [99], while the MAP kinase BcSAK1 – which becomes activated in response to oxidative stress – is required for conidiogenesis [100]. These oxygen species also play a fundamental signaling role in *N. crassa*, including cell differentiation. Thus, NOX-1 is required for both sexual and asexual development, such that Δ*nox-1* mutants are unable to differentiate mature fruiting bodies, producing a reduced number of conidia [101]. Moreover, in *N. crassa*, the increase of ROS (by the addition of menadione, or deletion of *sod-1*) allows to visualize in race tubes the rhythmic output of the circadian clock on the control of conidiation [102]. Therefore, it is tempting to speculate that the enhanced “banding” phenotype observed under 12:12 LD photocycles for the Δ*bcwcl1* mutant may be, at least partially explained, by differences in the intracellular ROS levels. As a matter of fact, at least on race tubes, these bands are reduced in the presence of an antioxidant (data not shown). While this cyclic banding can also be detected in constant darkness for *N. crassa* due to the existence of a circadian clock, the absence of an overt conidiation rhythm in *B. cinerea* under DD conditions is not evidence for lack of circadian regulation in this latter organism (Canessa et al., unpublished observations).

*B. cinerea* does sense light during infection resulting in increased expression levels of photoreceptor-encoding genes (data not shown). Therefore, it is reasonable to hypothesize a role of light perception in modulating the interaction of *B. cinerea* and its host. For this latter autotrophic organism, light has an outstanding relevance as it uses it as the energy source. A complex photoreceptor network comprising cryptochromes, blue light-sensing phototropins and phytochromes is present in plants regulating growth and stomata closure [103] but also defense responses to abiotic and biotic stresses. For instance, UV light increases resistance of *A. thaliana* to *B. cinerea* inoculated after the UV treatment [104], while a reduced red/far-red light ratio enhances susceptibility [105], [106]. Contrasting to the findings in *A. thaliana*, red light treatment induces resistance of *Vicia faba* (broad bean) [107] indicating that light admittedly increases resistance to *B. cinerea* but the effective wavelengths may differ in distinct plant species.

The results presented here exemplify how light can modify morphogenesis and pathogenicity in *B. cinerea*. Due to the problems arising from the host responses and intrinsic physiology, the effect of light on the potential of *B. cinerea* to cause disease is difficult to address. Consequently, the use of partially blind mutants may contribute to the understanding of the importance of light during the fungal-plant interaction from the fungal perspective. Here we demonstrate a function for BcWCL1 during plant infection in the presence of light, as reduced lesion sizes were observed in the Δ*bcwcl1* mutant, in comparison with the wild-type strain, being this effect more dramatic under constant light conditions than in photocycles. Since excessive light impairs the growth of the mutant by generating ROS, the Δ*bcwcl1* mutant may have problems to cope with ROS that are produced by the plant within the extent of an oxidative burst as part of the host defense mechanisms, subsequently hampering the fungal ability to colonize the plant tissue. In this regard, studies performed in *A. thaliana* have reported on the capacity of light to elevate the H_2_O_2_ production, with the concomitant increased callose formation [108]. In addition, Islam & co-workers [107] reported on positive and negative phototropism of germ tubes on onion and broad bean epidermal strips. While long-wave light treatments resulted in positive responses and elongated non-penetrating hyphae, negative phototropism and infection hyphae formation was observed in response to short-wave light. Under this premise, it is reasonable to hypothesize that the absence of BcWCL1 may affect the differentiation of infection structures.

In aggregate, we have provided molecular evidence of transcriptional responses to light in *B. cinerea* of which some, but not all, depend on a WC-1 ortholog. Clear responses to light are enhanced in the absence of the WCC revealing a more complex dialogue between this and other photoreceptors. Moreover, BcWCL1 is important – in the light – highlighting the role of light sensing in *B. cinerea* physiology and virulence.

## Supporting Information

Figure S1 **Genotypification of Δ** ***bcwcl1*** **strains.** (**A**) Replacement strategies showing the used replacement cassettes and the expected *in-locus* insertion of constructs. Schematic representation of a 10.5 kb genomic region (between BamHI restriction sites) of the *bcwcl1* locus (located in B05.10 Supercontig 119, Broad Database). *Bcwcl1* and its transcriptional orientation (3,765 bp; Bc1G_13505, genomic coordinates 27645–31409) is represented as a blue arrow. A single intron, located towards the 3′-end of the gene, is indicated in a white box. Gene model Bc1G_13504 is shown as a reference. The gene replacements cassettes employed to obtain Δ*bcwcl1* (mutant 1) and Δ*bcwcl1* (mutants 2 and 3) strains are shown above and below, respectively. In both cases, the position of the genomic regions employed for the homologous recombination (orange boxes) and KO generation are shown (to scale) next to *bcwcl1*. Gene model Bc1G_13506 (located downstream the 3′-flank) has been omitted from the scheme. Black arrows show primers used for diagnostic PCRs (Table S2), indicating their respective position and orientation. (**B**) Diagnostic PCRs. Homologous integration at 5′- and 3′- regions are shown for all mutants. No wild-type (WT) alleles were observed in Δ*bcwcl1* mutants after single-spore isolation (see methods) in comparison with the wild-type strain (B05.10). Primer pairs, and their corresponding sequences, are indicated in Table S2. (**C**) Southern blot hybridization. 10 µg of genomic DNA was digested with BamHI, and hybridized with the full-length *hph* CDS (expected sizes: mutant 1, 6,321 bp; mutants 2 and 3, 3,344 bp). To simplify the figure, only the hybridizations of mutants 1 and 2 are shown (lanes 1 and 2, respectively).
(TIF)

Figure S2 **The osmolarity of the medium affects sclerotia formation in DD.** (**A**) The nutritional status modulates light-dependent differentiation. Strain B05.10 was cultivated on solid media during 14 d in LD (upper panel) or DD (lower panel). MM (minimal medium), CM (complete medium), complex media containing plant components: PDAB (potato dextrose agar supplemented with instant mashed potatoes and pureed bean leaves), V8 (diluted vegetable juice) and GJ (undiluted grape juice). (**B**) High osmolarities prevent sclerotial development in DD. Strain B05.10 was cultivated during 14 d in DD on supplemented CM as indicated in the figure.
(TIF)

Figure S3 **Phylogenetic trees of** ***white collar*** **TFs from selected ascomycetes.** Schematic representation of BcWCL1 (**A**) and BcWCL2 (**B**) proteins. Protein domains and nuclear localization signals (NLS, indicated in yellow) were predicted by Pfam (http://pfam.sanger.ac.uk) and WoLF PSORT (http://wolfpsort.org). LOV: light-oxygen-voltage domain; PAS: PER-ARNT-SIM domain; ZN: GATA-type zinc finger DNA-binding domain. Sequence alignments and tree constructions were performed using the “One Click” method and standard parameters at Phylogeny.fr (http://www.phylogeny.fr). Orthologs from the basidiomycete *Coprinopsis cinerea* were employed as outgroups. Protein accession numbers of BcWCL1 orthologs are: *S. sclerotiorum* (SS1G_11953, SS1G_11954, revised annotation), *N. crassa* WC-1 (NCU02356.7), *Trichoderma reesei* (AAV80185.1), *Fusarium fujikuroi* WcoA (CAO85915.1), *Magnaporthe oryzae* MGWC1 (MGG_03538.5), *A. nidulans* LreA (CBF82714.1), *A. fumigatus* LreA (EAL92988.1) and *C. cinerea* DST1 (BAD99145.1). Protein accession numbers of BcWCL2 orthologs are: *S. sclerotiorum* (SS1G_12238), *N. crassa* WC-2 (NCU00902.7), *T. reesei* (AAV80186.1), *Fusarium verticillioides* (ADG85115.1), *M. oryzae* (MGG_04521), *A. nidulans* LreB (AAP47576.1), *A. fumigatus* LreB (XP_751563.1) and *C. cinerea* (BAK82128.1).
(TIF)

Figure S4 **Red light promotes “banding” phenotype of B05.10 and Δ** ***bcwcl1*** **.** Strains were grown in race tubes under LD conditions. Representative pictures were acquired after 14 d of incubation from the top and bottom section of each tube. (**A**) Full-spectrum white light. (**B**) Red light was generated using a pale yellow-light filter (deep straw, transmission percentage of over 51% for λ = 540 nm and over).
(TIF)

Figure S5 **Light and its absence still affect conidiation in** ***bcwcl1*** **deletion mutants.** (**A**) The initiation of conidiation in Δ*bcwcl1* occurs in a light-dependent fashion. Both B05.10 and Δ*bcwcl1* strains were incubated during 4 d in LL, LD or DD. (**B**) “Banding” phenotype of Δ*bcwcl1* mutant is light-dependent. Strains were grown for 7 d on solid CM. Addition of 0.02% SDS (indicated with asterisks; lower panel) results in comparably reduced daily growth rates for both strains, illustrating the “banding” in response to LD cycles. Strains reached the edges of the Petri dishes after 3 and 5 d of incubation (CM or CM + SDS, respectively).
(TIF)

Figure S6 **Complementation of the** ***bcwcl1*** **deletion mutant.** (**A**) Genotypification of the Δ*bcwcl1* complemented strain (Δ*bcwcl1+bcwcl1*) showing the amplification of *bcwcl1* (*bcwcl1*-ORF; oL586+ oL587) inserted at the *bcniaD* locus (*bcniaD*-5′(*nat1*); oL1226+ oL1716) and not at the *bcwcl1* locus (*bcwcl1*-3′; oL1226+ oL589), which contains the *hph* cassette used for *bcwcl1* deletion (*bcwcl1*-5′ (*hph*); oL588+ oL585). Primer pairs are indicates in Table S2. (**B**) Phenotypic characterization of a representative Δ*bcwcl1+bcwcl1* complemented strain demonstrated the restoration of sclerotia formation under DD culture conditions. (**C**) RT-qPCR of the Δ*bcwcl1+bcwcl1* mutant showing restoration of light-inducibility of gene expression (DD: constant darkness; LP: 60 min light pulse). Values are referred to the B05.10 strain grown under DD conditions (control  = 1). Bars represent mean values ± SEM. *Bcfrq1* and *bcvvd1* were chosen since no light-mediated transcriptional responses are observed in the Δ*bcwcl1* strain.
(TIF)

Figure S7 **No differences were observed for H_2_O_2_ accumulation in B05.10- and Δ** ***bcwcl1*** **-infected plant tissues.** *A. thaliana* Col-0 plants were inoculated with conidial suspensions of the indicated strains and incubated in LL, LD or DD conditions. After 3 d, leaves were detached and subjected to 3,3′-diaminobenzidine (DAB) staining. A brown precipitate, indicative for H_2_O_2_ accumulation was observed in infected but not in non-inoculated plant tissues (data not shown). Scale bars represent 500 µm.
(TIF)

Table S1 Oligonucleotides employed in RT-qPCR analysis. The table shows each amplified gene, including amplicons size, RT-qPCR dynamic range and RT-qPCR efficiency and related parameters. (FW: forward orientation; RC: reverse orientation). Gene IDs of the B05.10 strain annotation are indicated (Broad Database). * For these primers, an annealing temperature of 60°C was employed.
(DOCX)

Table S2 Oligonucleotides employed for generation of *bcwcl1* replacement and complementation cassettes. The table shows the oligonucleotides employed for each genetic construct (see Supplementary Figure S1A), used to obtain the vector employed for the generation of Δ*bcwcl1* mutant 1 (replacement cassette A), the second vector employed in Δ*bcwcl1* mutants 2 and 3 (replacement cassette B) and the Δ*bcwcl1* complementation vector (Figure S6). The table also indicates primer pairs employed for diagnostic PCRs (Figure S1B and S6A; FW: forward orientation; RC: reverse orientation). Overlapping regions are indicated in bold type, while those overlapping the pRS426 vector sequences are underlined.
(DOCX)
