# Supplementary figures and images for: Assessing the Effects of Light on Differentiation and Virulence of the Plant Pathogen Botrytis cinerea: Characterization of the White Collar Complex
Source: PLoS One. 2013 Dec 31;8(12):e84223. doi: 10.1371/journal.pone.0084223 (PMC3877267; doi:10.1371/journal.pone.0084223)

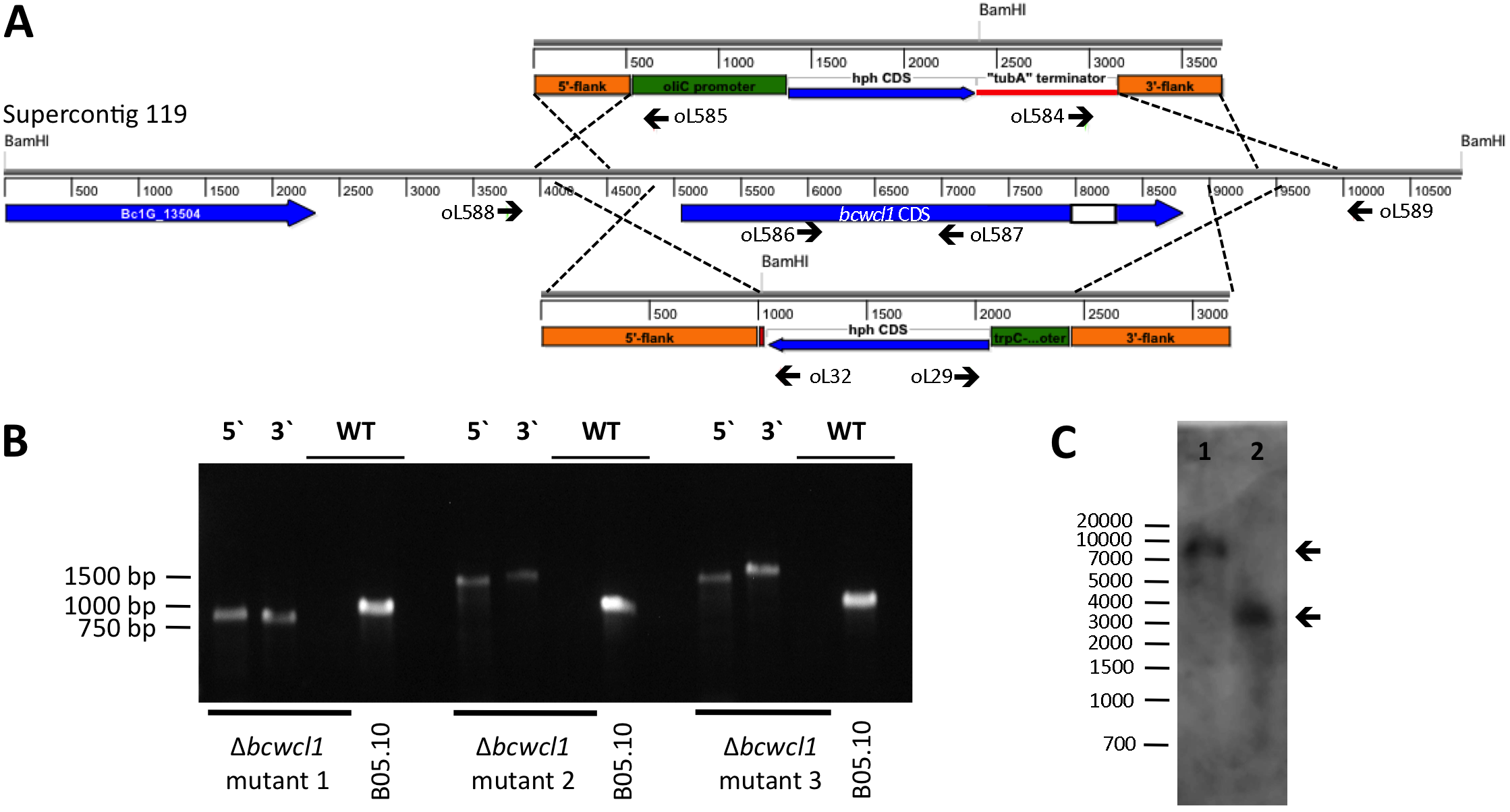

Supplement: Figure S1 — Genotypification of Δ bcwcl1 strains. (A) Replacement strategies showing the used replacement cassettes and the expected in-locus insertion of constructs. Schematic representation of a 10.5 kb genomic region (between BamHI restriction sites) of the bcwcl1 locus (located in B05.10 Supercontig 119, Broad Database). Bcwcl1 and its transcriptional orientation (3,765 bp; Bc1G_13505, genomic coordinates 27645–31409) is represented as a blue arrow. A single intron, located towards the 3′-end of the gene, is indicated in a white box. Gene model Bc1G_13504 is shown as a reference. The gene replacements cassettes employed to obtain Δbcwcl1 (mutant 1) and Δbcwcl1 (mutants 2 and 3) strains are shown above and below, respectively. In both cases, the position of the genomic regions employed for the homologous recombination (orange boxes) and KO generation are shown (to scale) next to bcwcl1. Gene model Bc1G_13506 (located downstream the 3′-flank) has been omitted from the scheme. Black arrows show primers used for diagnostic PCRs (Table S2), indicating their respective position and orientation. (B) Diagnostic PCRs. Homologous integration at 5′- and 3′- regions are shown for all mutants. No wild-type (WT) alleles were observed in Δbcwcl1 mutants after single-spore isolation (see methods) in comparison with the wild-type strain (B05.10). Primer pairs, and their corresponding sequences, are indicated in Table S2. (C) Southern blot hybridization. 10 µg of genomic DNA was digested with BamHI, and hybridized with the full-length hph CDS (expected sizes: mutant 1, 6,321 bp; mutants 2 and 3, 3,344 bp). To simplify the figure, only the hybridizations of mutants 1 and 2 are shown (lanes 1 and 2, respectively). (TIF) [file pone.0084223.s001.tif]

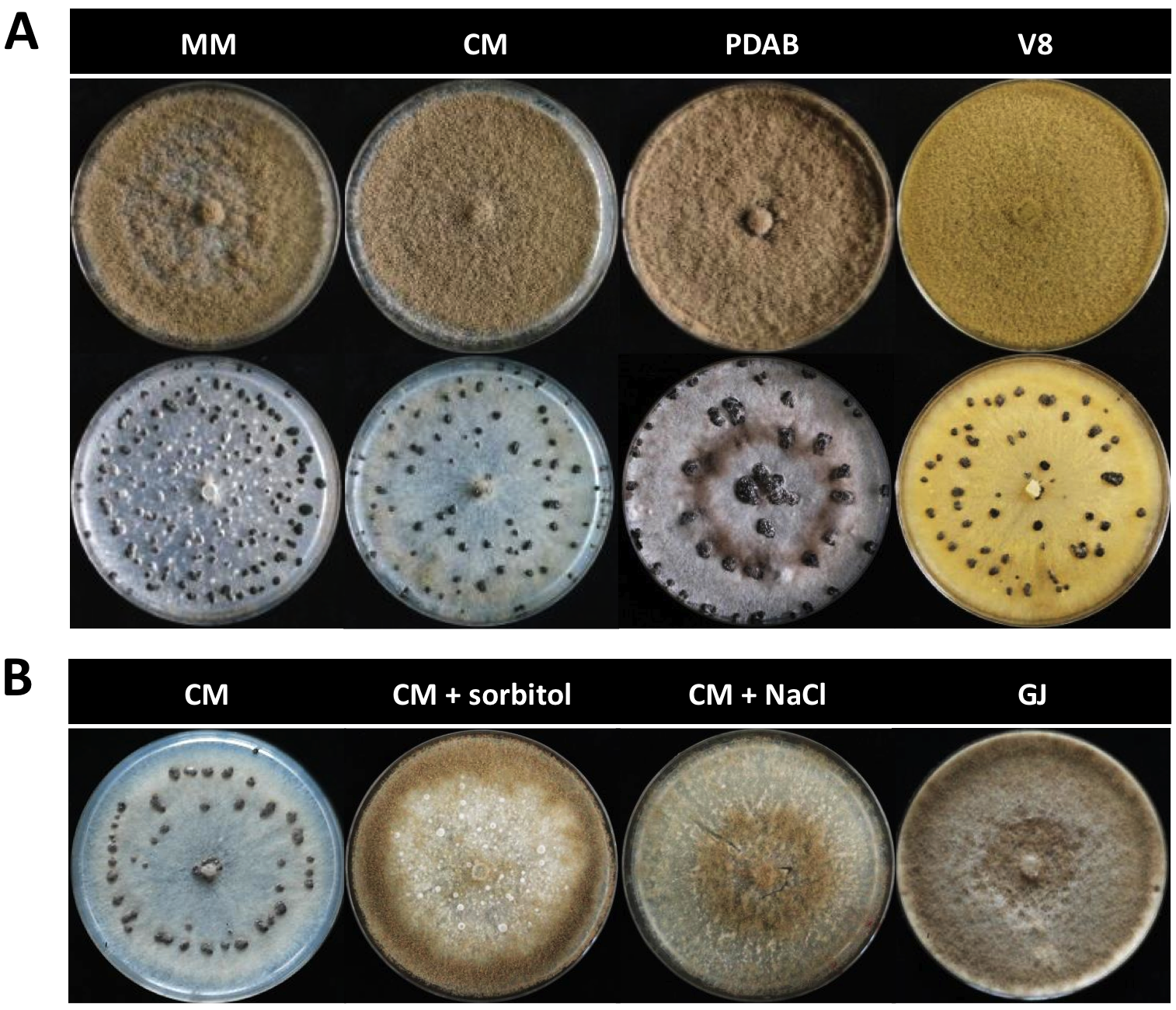

Supplement: Figure S2 — The osmolarity of the medium affects sclerotia formation in DD. (A) The nutritional status modulates light-dependent differentiation. Strain B05.10 was cultivated on solid media during 14 d in LD (upper panel) or DD (lower panel). MM (minimal medium), CM (complete medium), complex media containing plant components: PDAB (potato dextrose agar supplemented with instant mashed potatoes and pureed bean leaves), V8 (diluted vegetable juice) and GJ (undiluted grape juice). (B) High osmolarities prevent sclerotial development in DD. Strain B05.10 was cultivated during 14 d in DD on supplemented CM as indicated in the figure. (TIF) [file pone.0084223.s002.tif]

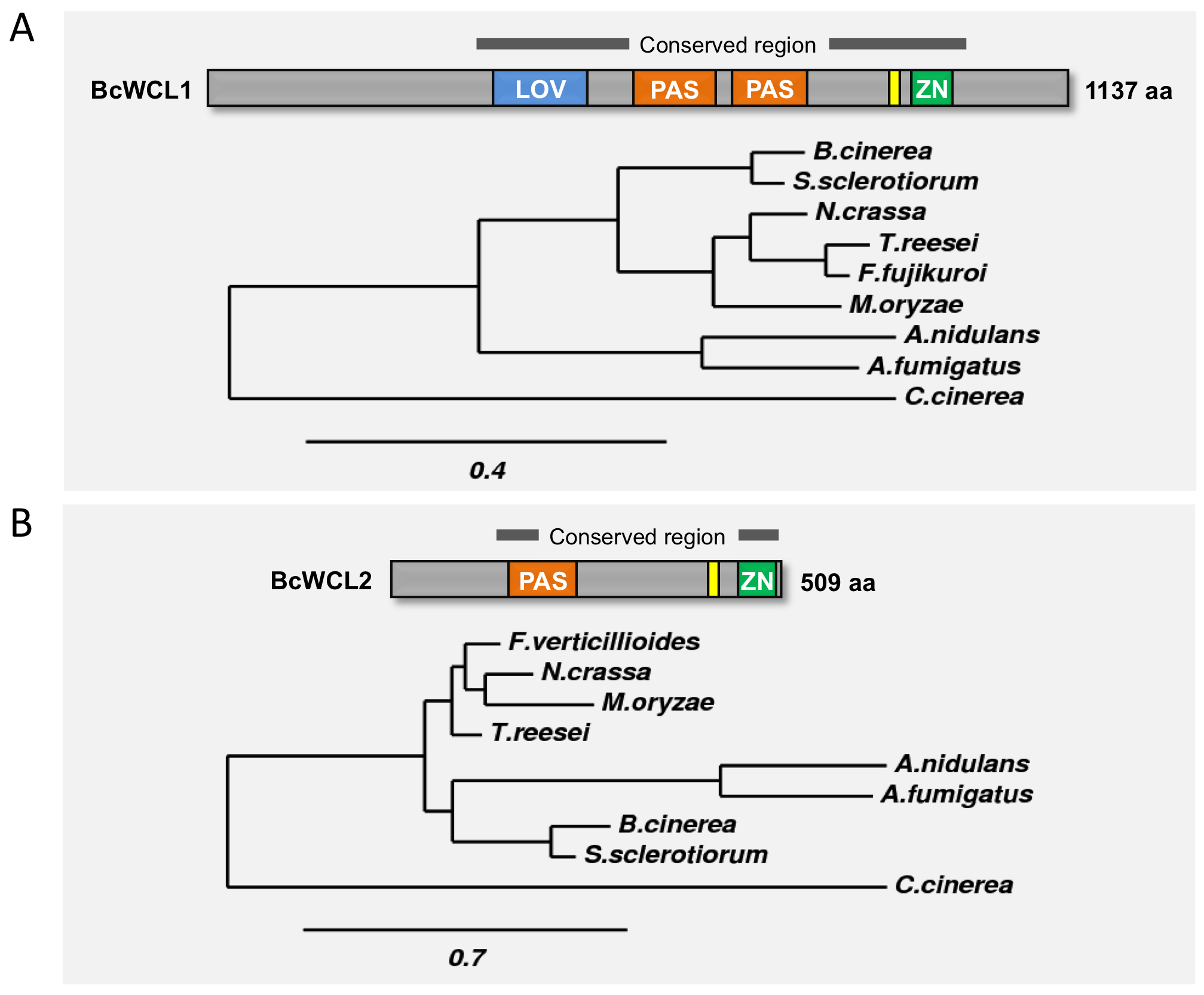

Supplement: Figure S3 — Phylogenetic trees of white collar TFs from selected ascomycetes. Schematic representation of BcWCL1 (A) and BcWCL2 (B) proteins. Protein domains and nuclear localization signals (NLS, indicated in yellow) were predicted by Pfam (http://pfam.sanger.ac.uk) and WoLF PSORT (http://wolfpsort.org). LOV: light-oxygen-voltage domain; PAS: PER-ARNT-SIM domain; ZN: GATA-type zinc finger DNA-binding domain. Sequence alignments and tree constructions were performed using the “One Click” method and standard parameters at Phylogeny.fr (http://www.phylogeny.fr). Orthologs from the basidiomycete Coprinopsis cinerea were employed as outgroups. Protein accession numbers of BcWCL1 orthologs are: S. sclerotiorum (SS1G_11953, SS1G_11954, revised annotation), N. crassa WC-1 (NCU02356.7), Trichoderma reesei (AAV80185.1), Fusarium fujikuroi WcoA (CAO85915.1), Magnaporthe oryzae MGWC1 (MGG_03538.5), A. nidulans LreA (CBF82714.1), A. fumigatus LreA (EAL92988.1) and C. cinerea DST1 (BAD99145.1). Protein accession numbers of BcWCL2 orthologs are: S. sclerotiorum (SS1G_12238), N. crassa WC-2 (NCU00902.7), T. reesei (AAV80186.1), Fusarium verticillioides (ADG85115.1), M. oryzae (MGG_04521), A. nidulans LreB (AAP47576.1), A. fumigatus LreB (XP_751563.1) and C. cinerea (BAK82128.1). (TIF) [file pone.0084223.s003.tif]

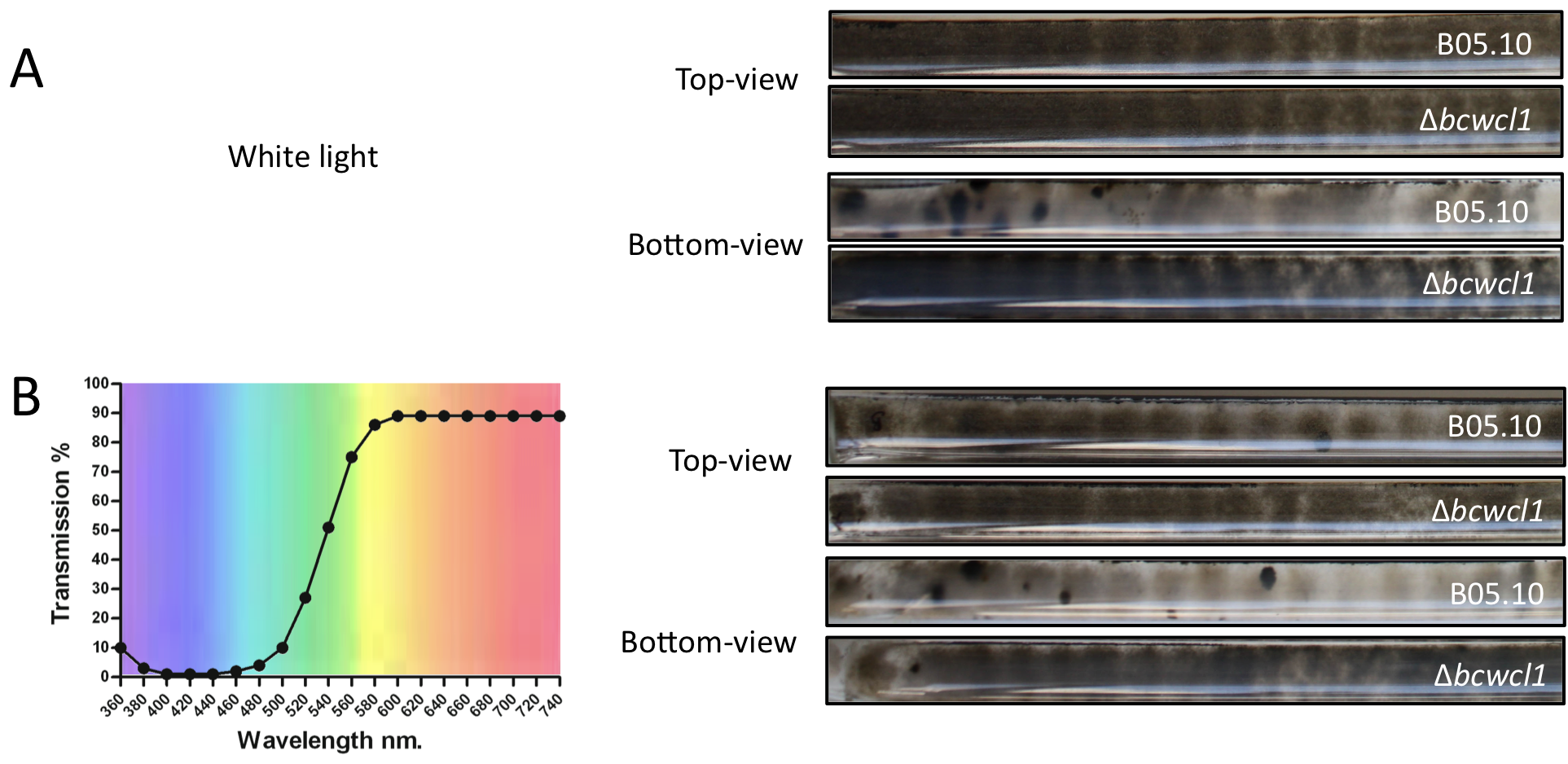

Supplement: Figure S4 — Red light promotes “banding” phenotype of B05.10 and Δ bcwcl1 . Strains were grown in race tubes under LD conditions. Representative pictures were acquired after 14 d of incubation from the top and bottom section of each tube. (A) Full-spectrum white light. (B) Red light was generated using a pale yellow-light filter (deep straw, transmission percentage of over 51% for λ = 540 nm and over). (TIF) [file pone.0084223.s004.tif]

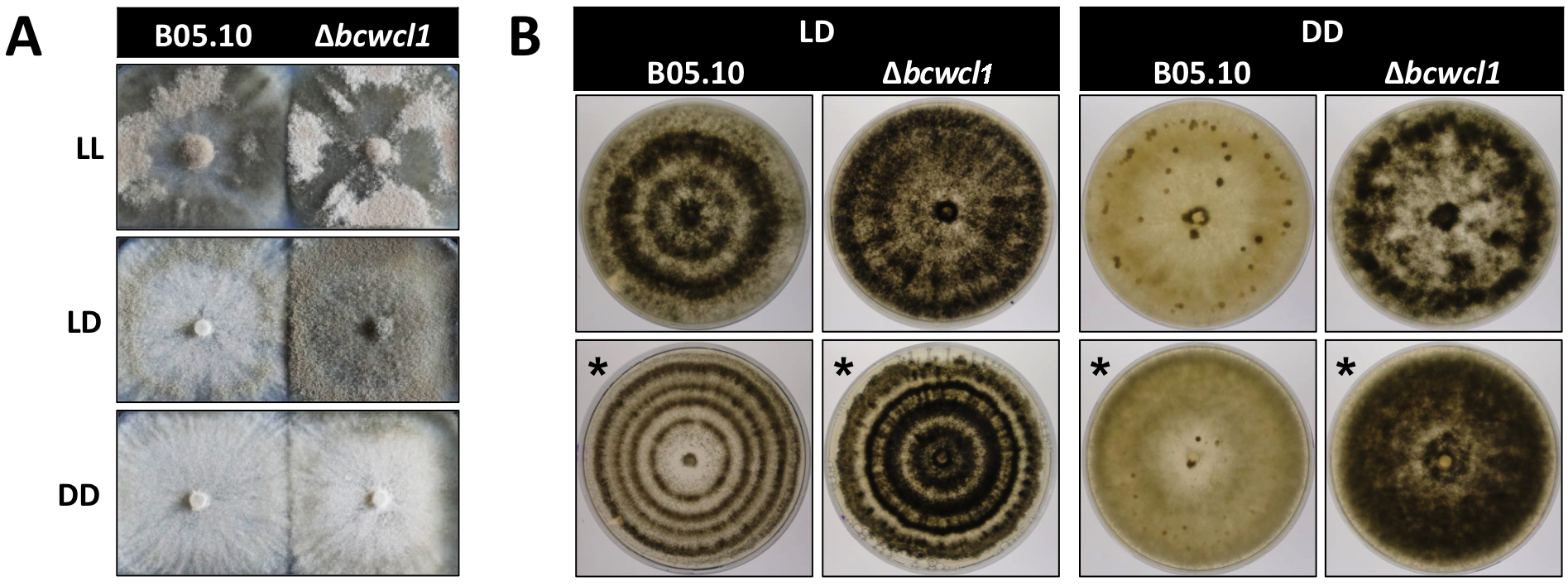

Supplement: Figure S5 — Light and its absence still affect conidiation in bcwcl1 deletion mutants. (A) The initiation of conidiation in Δbcwcl1 occurs in a light-dependent fashion. Both B05.10 and Δbcwcl1 strains were incubated during 4 d in LL, LD or DD. (B) “Banding” phenotype of Δbcwcl1 mutant is light-dependent. Strains were grown for 7 d on solid CM. Addition of 0.02% SDS (indicated with asterisks; lower panel) results in comparably reduced daily growth rates for both strains, illustrating the “banding” in response to LD cycles. Strains reached the edges of the Petri dishes after 3 and 5 d of incubation (CM or CM + SDS, respectively). (TIF) [file pone.0084223.s005.tif]

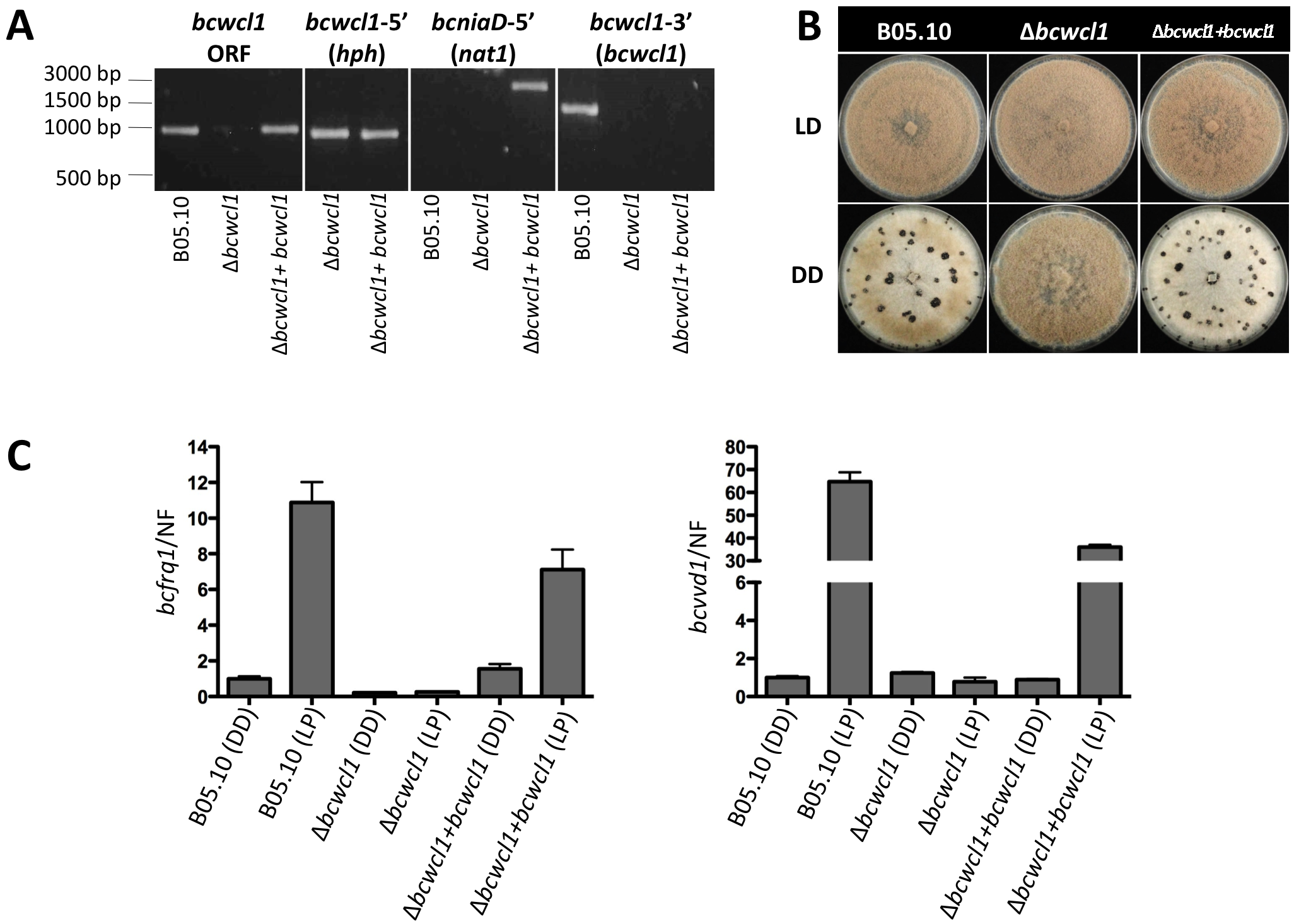

Supplement: Figure S6 — Complementation of the bcwcl1 deletion mutant. (A) Genotypification of the Δbcwcl1 complemented strain (Δbcwcl1+bcwcl1) showing the amplification of bcwcl1 (bcwcl1-ORF; oL586+ oL587) inserted at the bcniaD locus (bcniaD-5′(nat1); oL1226+ oL1716) and not at the bcwcl1 locus (bcwcl1-3′; oL1226+ oL589), which contains the hph cassette used for bcwcl1 deletion (bcwcl1-5′ (hph); oL588+ oL585). Primer pairs are indicates in Table S2. (B) Phenotypic characterization of a representative Δbcwcl1+bcwcl1 complemented strain demonstrated the restoration of sclerotia formation under DD culture conditions. (C) RT-qPCR of the Δbcwcl1+bcwcl1 mutant showing restoration of light-inducibility of gene expression (DD: constant darkness; LP: 60 min light pulse). Values are referred to the B05.10 strain grown under DD conditions (control = 1). Bars represent mean values ± SEM. Bcfrq1 and bcvvd1 were chosen since no light-mediated transcriptional responses are observed in the Δbcwcl1 strain. (TIF) [file pone.0084223.s006.tif]

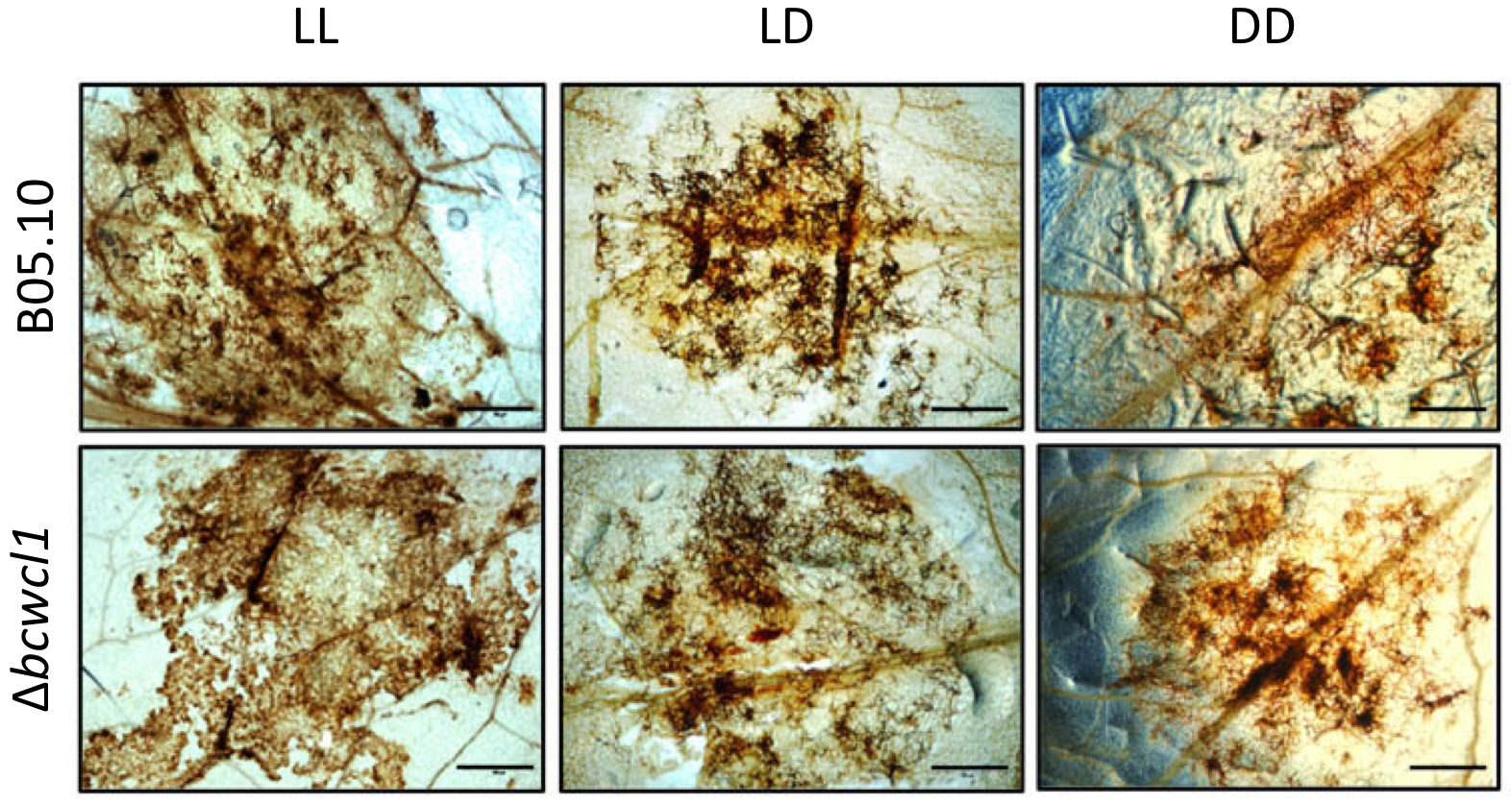

Supplement: Figure S7 — No differences were observed for H2O2 accumulation in B05.10- and Δ bcwcl1 -infected plant tissues. A. thaliana Col-0 plants were inoculated with conidial suspensions of the indicated strains and incubated in LL, LD or DD conditions. After 3 d, leaves were detached and subjected to 3,3′-diaminobenzidine (DAB) staining. A brown precipitate, indicative for H2O2 accumulation was observed in infected but not in non-inoculated plant tissues (data not shown). Scale bars represent 500 µm. (TIF) [file pone.0084223.s007.tif]
