# Supplementary material for: Assessing the Effects of Light on Differentiation and Virulence of the Plant Pathogen Botrytis cinerea: Characterization of the White Collar Complex
Source: PLoS One. 2013 Dec 31;8(12):e84223. doi: 10.1371/journal.pone.0084223 (PMC3877267; doi:10.1371/journal.pone.0084223)
Supplement: Table S1 — Oligonucleotides employed in RT-qPCR analysis. The table shows each amplified gene, including amplicons size, RT-qPCR dynamic range and RT-qPCR efficiency and related parameters. (FW: forward orientation; RC: reverse orientation). Gene IDs of the B05.10 strain annotation are indicated (Broad Database). * For these primers, an annealing temperature of 60°C was employed. (DOCX) [file pone.0084223.s008.docx]

**Table S1**

| **Gene** | **Primers 5’-3’** | **Primer’s name** | **Amplicon size (bp)** | **Cp lower level** | **Cp higher level** | **Efficiencies (%)** | **Efficiency curve Rsq** | **Efficiency curve order of magnitude** |
| --- | --- | --- | --- | --- | --- | --- | --- | --- |
| *bc actin*  BC1G_08198 | AGCGTGAAATCGTCCGTGAT(FW)  GACTGGCGGTTTGGATTTCTT(RC) | oL93  oL94 | 81 | 14.51 | 38.23 | 99.7 | 0.999 | 8 |
| *bc tubulin*  BC1G_05600 | GCCGGGTTGCCGATAATT(FW)  AGAAGAGCACCGAAACCAGATC(RC) | oL95  oL96 | 91 | 14.59 | 35.72 | 95.3 | 0.998 | 8 |
| *bcef1b*  BC1G_03337 | GGCTACCTTGAACCCAGCAA(FW)  AGCCTCCGCATCTTCTTCCT(RC) | oL297  oL298 | 100 | 11.73 | 24.19 | 106.6 | 0.997 | 5 |
| *bcwcl1*  BC1G_13505 | TCAATCAGTTCGTCCCCAAG(FW)  CACCTGGTTGCGTGATAGGT(RC) | oL245  oL246 | 92 | 18.36 | 35.39 | 102.6 | 0.994 | 6 |
| *bcwcl2*  BC1G_01840 | TCCGAACCATCCTGATTTCC(FW)  GATTCGCTTTCGTCAACGTG(RC) | oL325  oL326 | 80 | 11.84 | 32.29 | 95.88 | 0.999 | 7 |
| *bcfrq1*  BC1G_13940 | ACCCAGGAGGAAAGGTACGAA(FW)  GGGAGCGGAAGGACAGATTT(RC) | oL97  oL98 | 86 | 15.98 | 35.69 | 98.4 | 0.996 | 7 |
| BC1G_03545 | GGAAGCTTCGTCTGCAGGTC(FW)  GCTCTTTGTGCGGGAATGTT(RC) | oL291  oL292 | 120 | 11.96 | 25.65 | 95.8 | 0.999 | 5 |
| *bcltf1*  BC1G_10441 | ATGCGAAACTTACCCGCAAA(FW)  CTCCGAAGATTTGGGTCGAA(RC) | oL287  oL288 | 80 | 14.69 | 35.41 | 94.1 | 0.999 | 7 |
| *bccsp1*  BC1G_04022 | TGCCTGCCAGAGTACAAAAG(FW)  GAATGGAGGGATATCGGATG(RC) | oL1157 oL1158 | 104 | 8.91 | 31.48 | 96.8 | 0.992 | 8 |
| *bcadv1**  BC1G_05192 | TCGATTCGACGATTCCCTAC(FW)  AGGTCATGCGATTCGGTAAC(RC) | oL1493  oL1494 | 111 | 13.43 | 33.82 | 96.62 | 0.998 | 7 |
| *bcvad3**  Bc1G_03744 | GGTGGTTTCGACAAAGATCC(FW)  TACCATCTCTTTCGGCATGG(RC) | oL1497  oL1498 | 108 | 13.79 | 34.18 | 96.43 | 0.999 | 7 |
| *bcsah1**  Bc1G_08188 | ACGATGTACGATTCCGATGC(FW)  ACCGAACCGAAACTGCTATG(RC) | oL1487  oL1488 | 77 | 13.11 | 33.36 | 96.97 | 0.999 | 7 |
| *bcvvd1*  BC1G_04348 | CTTTGATTATCTCGGCAATGCA(FW)  AGCGCGTTTCGAATGGTTAA(RC) | oL99  oL100 | 96 | 15.88 | 36.96 | 91.2 | 0.998 | 7 |
| *bop1*  BC1G_02456 | GTCTGGGGTATTGGTGATGG(FW)  TACCGTCATGAGCGAACAAG(RC) | oL1171  oL1172 | 124 | 10.14 | 34.69 | 95.0 | 0.983 | 8 |
| *bccry1*  BC1G_13162 | TGATCGTCGGCTCTTTTCTC(FW)  CATCCCCCGTTATTACTTGC(RC) | oL1153  oL1154 | 112 | 11.17 | 35.00 | 93.0 | 0.987 | 8 |
| *bcccg1**  Bc1G_11685 | ATCCAAGGTGCCACTGCTAC(FW)  ACATCAGCCTTGGTGTTGTG(RC) | oL1482  oL1483 | 149 | 11.38 | 32.14 | 94.06 | 0.999 | 7 |
| *bcfer1*  BC1G*_*16428 | TCCAGTCTTCATCCAAGCAC(FW)  CAGCGCATTTCTCACTCTTG(RC) | oL1285  oL1286 | 116 | 12.34 | 31.41 | 83.3 | 0.919 | 6 |
