# Supplementary material for: Assessing the Effects of Light on Differentiation and Virulence of the Plant Pathogen Botrytis cinerea: Characterization of the White Collar Complex
Source: PLoS One. 2013 Dec 31;8(12):e84223. doi: 10.1371/journal.pone.0084223 (PMC3877267; doi:10.1371/journal.pone.0084223)
Supplement: Table S2 — Oligonucleotides employed for generation of bcwcl1 replacement and complementation cassettes. The table shows the oligonucleotides employed for each genetic construct (see Supplementary Figure S1A), used to obtain the vector employed for the generation of Δbcwcl1 mutant 1 (replacement cassette A), the second vector employed in Δbcwcl1 mutants 2 and 3 (replacement cassette B) and the Δbcwcl1 complementation vector (Figure S6). The table also indicates primer pairs employed for diagnostic PCRs (Figure S1B and S6A; FW: forward orientation; RC: reverse orientation). Overlapping regions are indicated in bold type, while those overlapping the pRS426 vector sequences are underlined. (DOCX) [file pone.0084223.s009.docx]

**Table S2**

| **Amplification target** | **Primers 5’-3’** | **Primer name** | **Strain** |
| --- | --- | --- | --- |
| *bcwcl1*  upstream non-coding region | **GCGGATAACAATTTCACACAGGAAACAGC**ACCAGCTGTGGAAGTGTCTG (FW) | LC-256 | ∆*bcwcl1* mutant 1 |
| *bcwcl1*  upstream non-coding region | **CGAATCGGGAATGCGGCTCCACAGCTGCAG**GGTATTTGAGCGGAGGCAGG (RC) | LC-276 | ∆*bcwcl1* mutant 1 |
| *bcwcl1*  downstream non-coding region | **TTAATGATGATTACTAACAGATATCAAGCTT**TGTCATGTTGTTATAGTCTCGG  (FW) | LC-277 | ∆*bcwcl1* mutant 1 |
| *bcwcl1*  downstream non-coding region | **GTAACGCCAGGGTTTTCCCAGTCACGACG**ATCTGTATCGGGGTTGTGTTT (RC) | LC-278 | ∆*bcwcl1* mutant 1 |
| *hph* resistance cassette (from pLOB1 vector) | CTGCAGCTGTGGAGCCGCATT(FW) | LC-269 | ∆*bcwcl1* mutant 1 |
| *hph* resistance cassette (from pLOB1 vector) | AAGCTTGATATCTGTTAGTAA(RC) | LC-270 | ∆*bcwcl1* mutant 1 |
| *bcwcl1*  upstream non-coding region | **GCGGATAACAATTTCACACAGGAAACAGC**AGGATATACCGAGGGACGAG (FW) | oligo1 | ∆*bcwcl1* mutants 2 & 3 |
| *bcwcl1*  upstream non-coding region | **GACCGGGATCCACTTAACGTTACTGAAATC**ATTGAAGAAATTACTCATGATA  (RC) | oligo2 | ∆*bcwcl1* mutants 2 & 3 |
| *bcwcl1*  downstream non-coding region | **CAAAAATGCTCCTTCAATATCATCTTCTGTC**TTGGCAGGGTTCTCCTGGAT (FW) | oligo3 | ∆*bcwcl1* mutants 2 & 3 |
| *bcwcl1*  downstream non-coding region | **GTAACGCCAGGGTTTTCCCAGTCACGACG**GTTGAATTGGAAAACTTGGA (RC) | oligo4 | ∆*bcwcl1* mutants 2 & 3 |
| *hph* resistance cassette (from pCSN44 vector) | GACAGAAGATGATATTGAAGGAGC (FW) | oL768 | ∆*bcwcl1* mutants 2 & 3 |
| *hph* resistance cassette (from pCSN44 vector) | GATTTCAGTAACGTTAAGTGG (RC) | oL687 | ∆*bcwcl1* mutants 2 & 3 |
| Amplification of genetic constructs (from pRS426) | GGCAGTGAGCGCAACGCAAT (FW) | oL83 | - |
| Amplification of genetic constructs (from pRS426) | ATTCAGGCTGCGCAACTGTT (RC) | oL84 | - |
| Amplification of *bcwcl1*, to be fused to P*oliC* promoter | TCCATCACATCACAATCGATCCAACCATGCCAATGACCCAAGCAGACTTG (FW) | *bcwcl1*-P*oliC*-F | ∆*bcwcl1* +*bcwcl1* |
| Amplification of *bcwcl1*, to be fused to T*gluc* terminator | AATCATACATCTTATCTACATACGCTAACCCTCCTTGAAAATCTCATTA (RC) | *bcwcl1*-T*gluc*-R | ∆*bcwcl1* +*bcwcl1* |
| 5’ integration, at *bcwcl1* locus; diagnostic PCR | GAGAGACAGGATGGTACAAATGAG (FW) | oL588 | ∆*bcwcl1* mutant 1, 2 & 3 |
| 3’ integration, at *bcwcl1* locus; diagnostic PCR | ACACTTGTGCTTGTGCTTGC (RC) | oL589 | ∆*bcwcl1* mutant 1, 2 & 3 |
| 5’ integration, at P*oliC* promoter; diagnostic PCR | GGTACTGCCCCACTTAGTGGCAGCTCGCG (RC) | oL585 | ∆*bcwcl1* mutant 1 |
| 3’ integration, at “*tubA*” terminator; diagnostic PCR | GGTCCTCGGAGTGCAGATGGG (FW) | oL584 | ∆*bcwcl1* mutant 1 |
| 5’ integration, at *hph* CDS; diagnostic PCR | ATGGCTGTGTAGAAGTACTC (FW) | oL32 | ∆*bcwcl1* mutants 2 & 3 |
| 3’ integration, at *hph* CDS; diagnostic PCR | TCGCCCTCCGAGAGCTGCAT (RC) | oL29 | ∆*bcwcl1* mutants 2 & 3 |
| *bcwcl1* ORF, diagnostic PCR | GTATCAACCCCCACGGCTCTCA (FW) | oL586 | - |
| *bcwcl1* ORF, diagnostic PCR | CGGCGAATGCGGAATACCA (RC) | oL587 | - |
| *bcwcl1* 5’ integration at *bcniaD* locus, diagnostic PCR | GGCATCTCTTGGAGGAAGAA (FW) | oL1226 | ∆*bcwcl1* +*bcwcl1* |
| 5’ integration at *bcniaD* locus, diagnostic PCR | CGCATATCAGCATATCGAGATGTCC (FW) | oL1716 | ∆*bcwcl1* +*bcwcl1* |
